# Supplementary material for: Segmented filamentous bacteria are worldwide human gut commensals
Source: Nat Commun. 2026 Mar 5;17:4174. doi: 10.1038/s41467-026-70010-4 (PMC13153320; doi:10.1038/s41467-026-70010-4)
Supplement: Supplementary file 1 — Supplementary Information [file 41467_2026_70010_MOESM1_ESM.pdf]

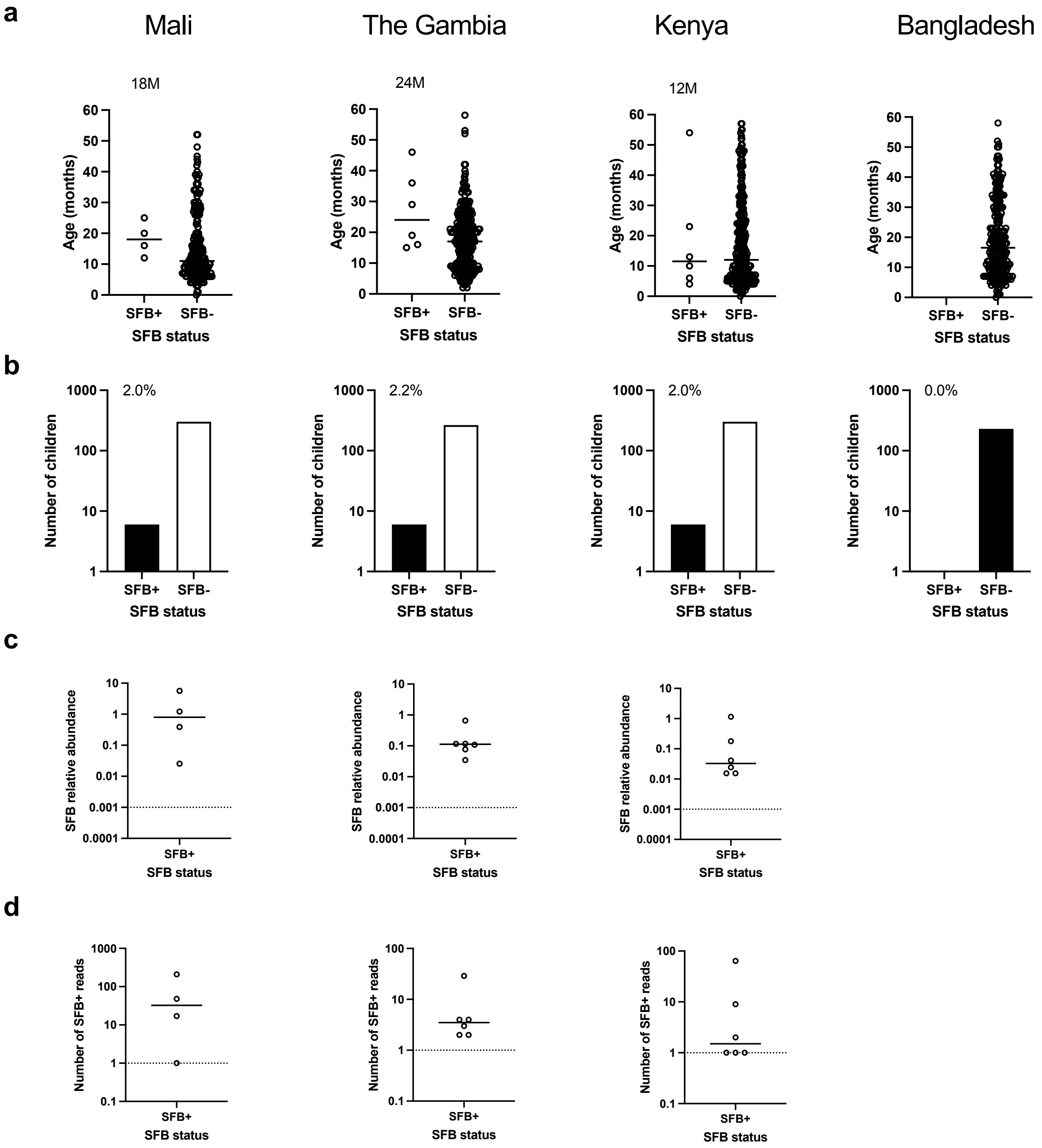

Supplementary Figure 1. Identification of SFB in a fecal 16S rRNA gene amplicon dataset. Analysis of fecal samples of bioproject PRJNA234437, a study of dysentery in children up to 5 years of age. Number of children: Mali n=307, The Gambia n=272, Kenya n=307, Bangladesh n=230. **a** Age distribution of fecal samples positive and negative for SFB and including the median age in months (M) of SFB-positive individuals. **b** SFB prevalence. **c** SFB relative abundance per sample. **d** SFB 16S rRNA gene read number per sample. **a,c/d** Crossbars indicate the median value of the dataset. **c/d** A dotted line is included **c** at a relative abundance of 0.001% and **d** at a read number of 1 or reference purposes.

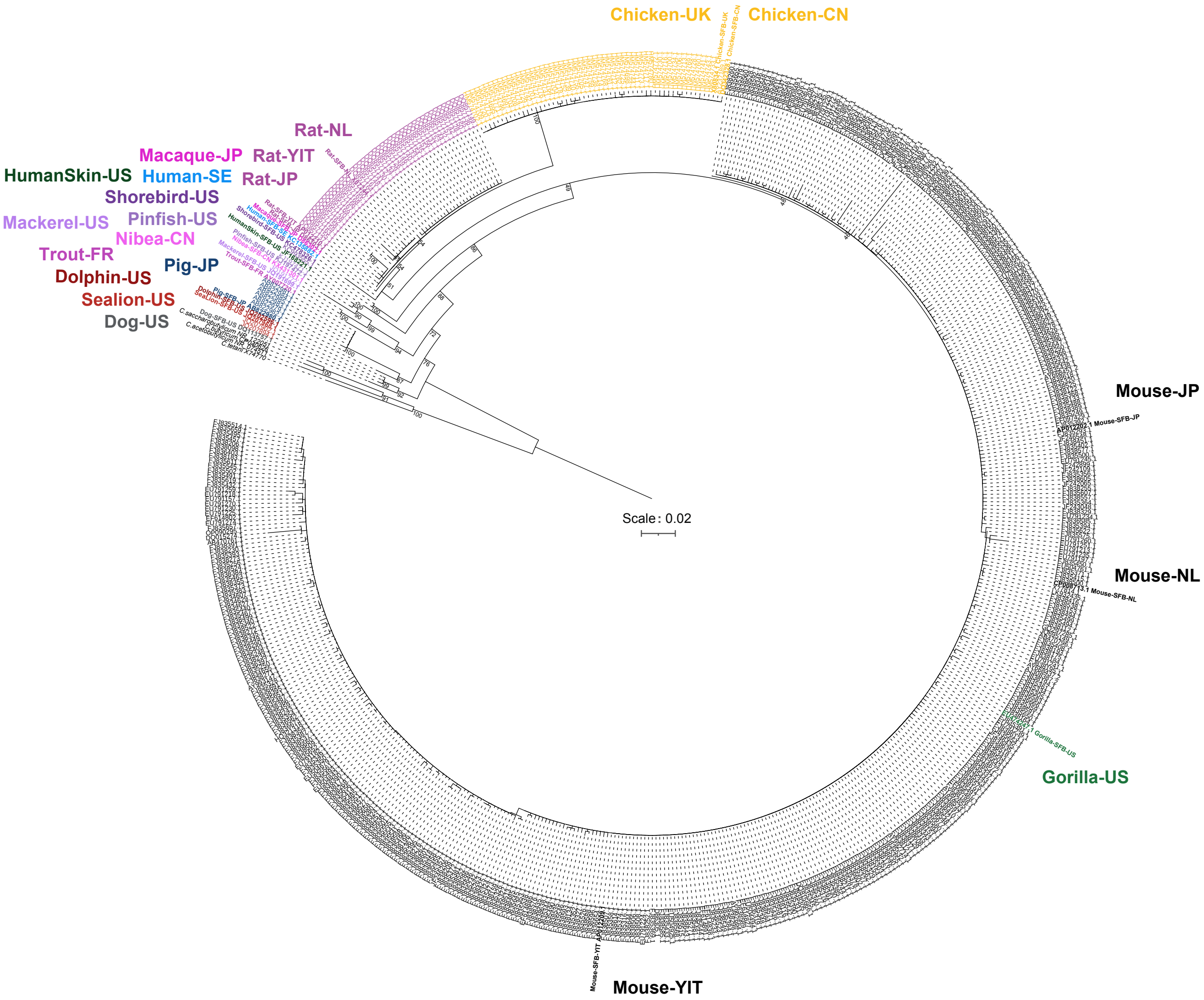

Supplementary Figure 2. Maximum likelihood phylogenetic tree of the 16S rRNA gene V1-V4 region for sequences available on the NCBI nt database that fall within the SFB clade. 16S rRNA gene sequences covering at least the V1-V4 region were identified in the NCBI nucleotide database by using the V1-V4 region of the 16S rRNA gene sequence from Mouse-SFB-NL as the search query. Reference sequences used for further analysis are highlighted in bold and their host and geographic origin, with the two-letter country code, are indicated. 16S rRNA gene sequences from the same host are colored according to the reference sequence. 16S rRNA gene sequences are labeled with their NCBI accession number. *Clostridium* outgroups are italicized. Tree includes bootstrap values and the scale is nucleotide substitutions per nucleotide position.

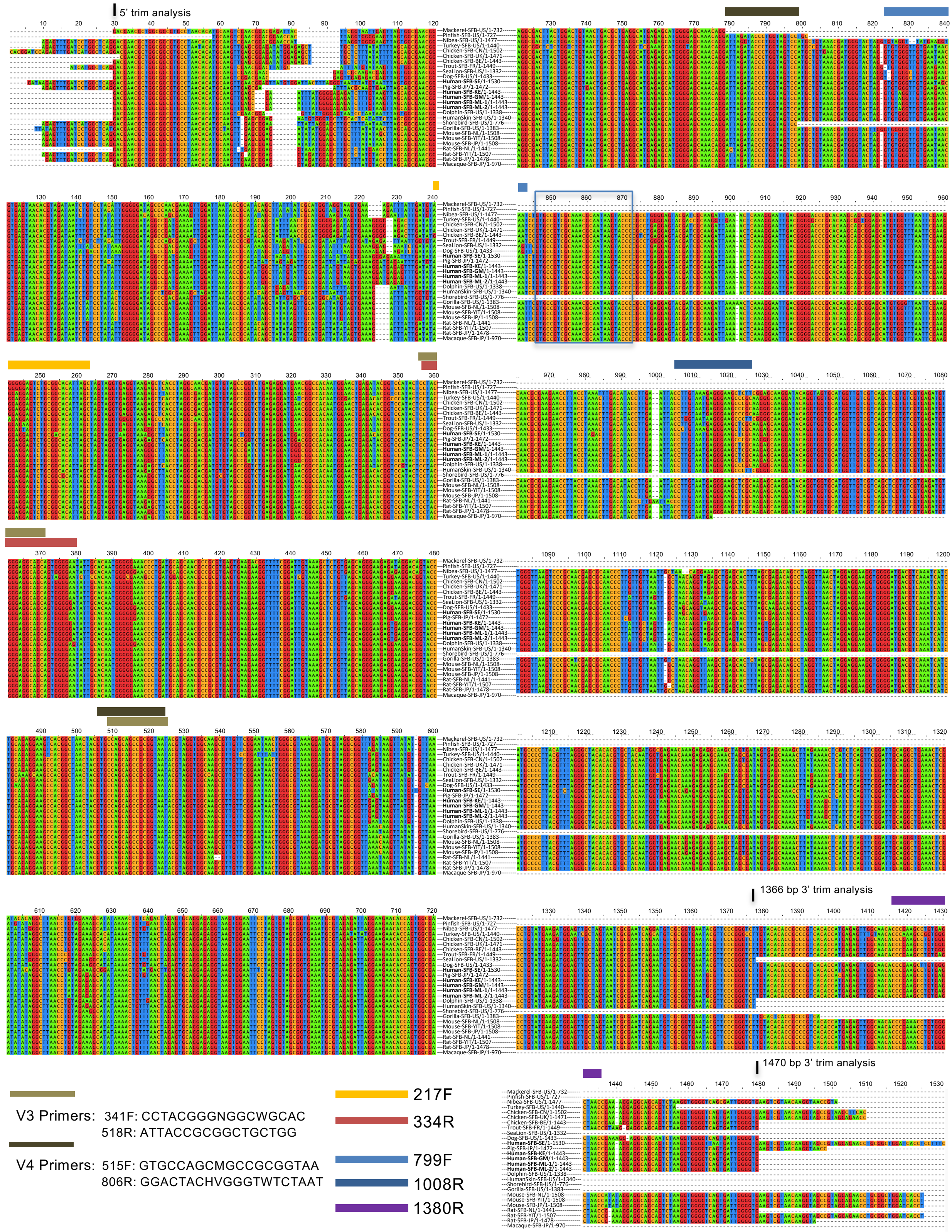

Supplementary Figure 3. Comparison of SFB reference 16S rRNA gene sequences from various host origins. Regions corresponding to common forward and reverse SFB primers used to detect SFB and primers used for general V3 and V4 region amplification purposes are included as a reference. Sequence complimentary to the SFB-specific probe (Urdaci *et al.*) used to detect SFB from various hosts by fluorescent *in situ* hybridization is highlighted with a box. Locations of the 5' and 3' terminus for sequence trimming before phylogenetic analysis are highlighted. Alignment performed with Clustal Omega and viewed with Jalview. The 16S rRNA gene sequence for Turkey-SFB-US (GCA_001655775.1) is derived from metagenomic data and is therefore not included in additional analyses. Nucleotides are color coded to increase clarity.

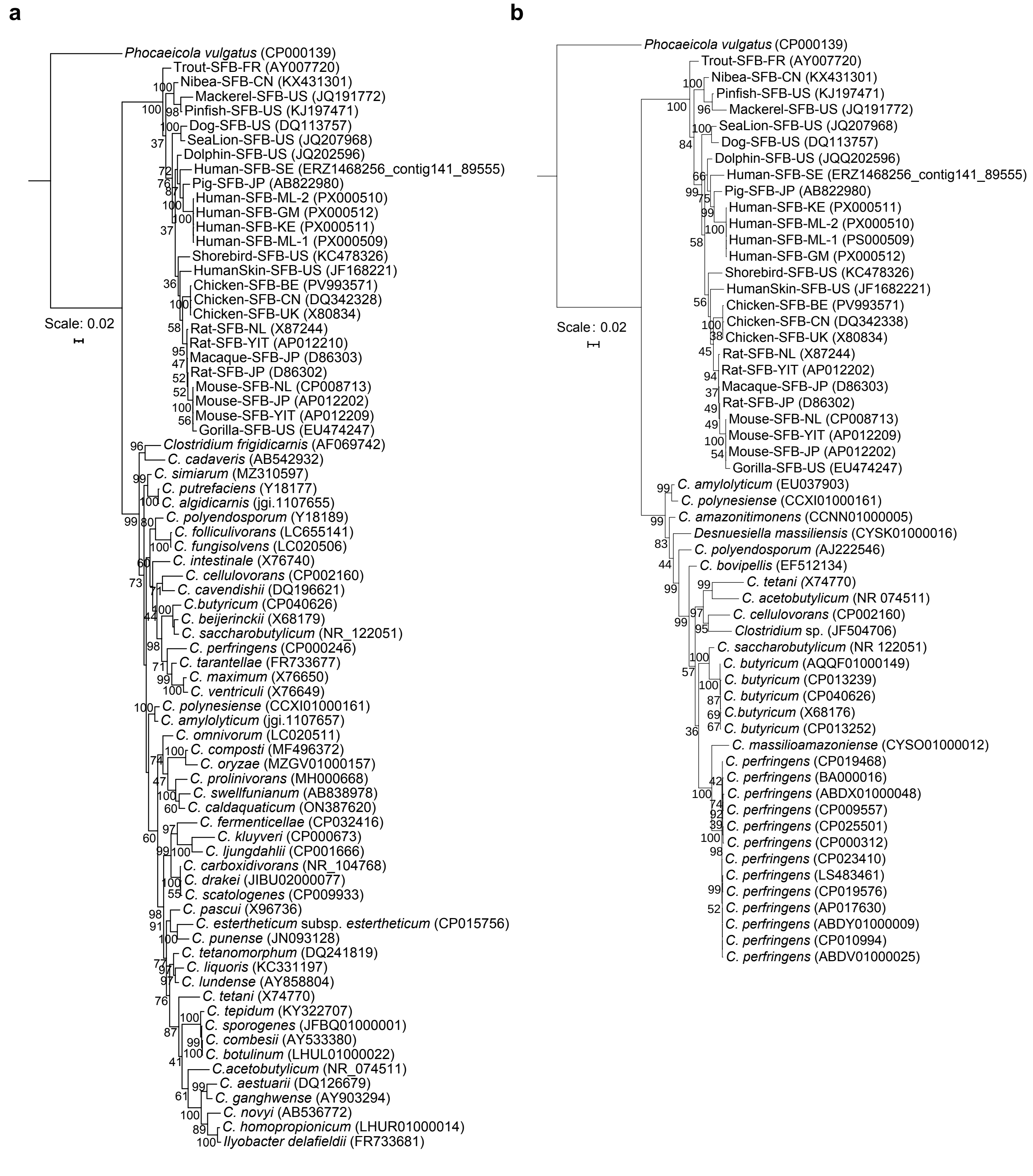

Supplementary Figure 4. Maximum likelihood phylogenetic trees of the 16S rRNA gene sequence from SFB of various hosts and the bacterial species most closely related to SFB. a/b. Maximum likelihood phylogenetic tree of SFB 16S rRNA gene reference sequences from various hosts, *Phocaeicola vulgatus* as the distantly related outgroup, and the most closely related bacterial 16S rRNA gene sequences from the **a** EzBioCloud database and the **b** Silva database. Sequences were trimmed to 1470 bp when possible (for exceptions see Supplementary Fig. 3). The sequences include their accession number and the trees include bootstrap values and a scale of nucleotide substitutions per nucleotide position.

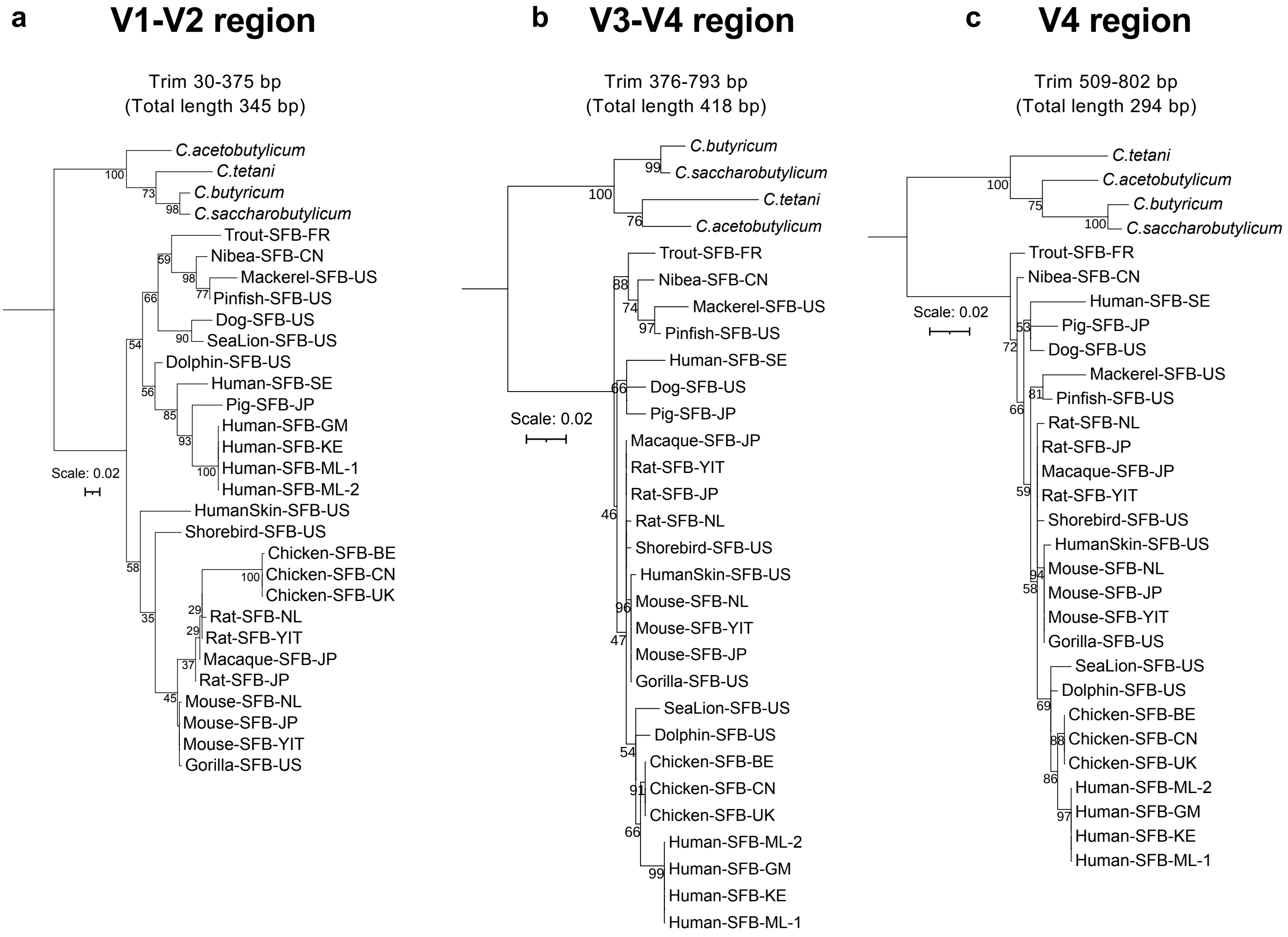

Supplementary Figure 5. Maximum likelihood phylogenetic trees for variable regions of the SFB 16S rRNA gene sequences from various hosts. Analysis of the **a** V1-V2; **b** V3-V4; and **c** V4 variable regions of SFB 16S rRNA gene reference sequences and including *Clostridium* outgroup species (italic). Trees include bootstrap values and the scale is nucleotide substitutions per nucleotide position.

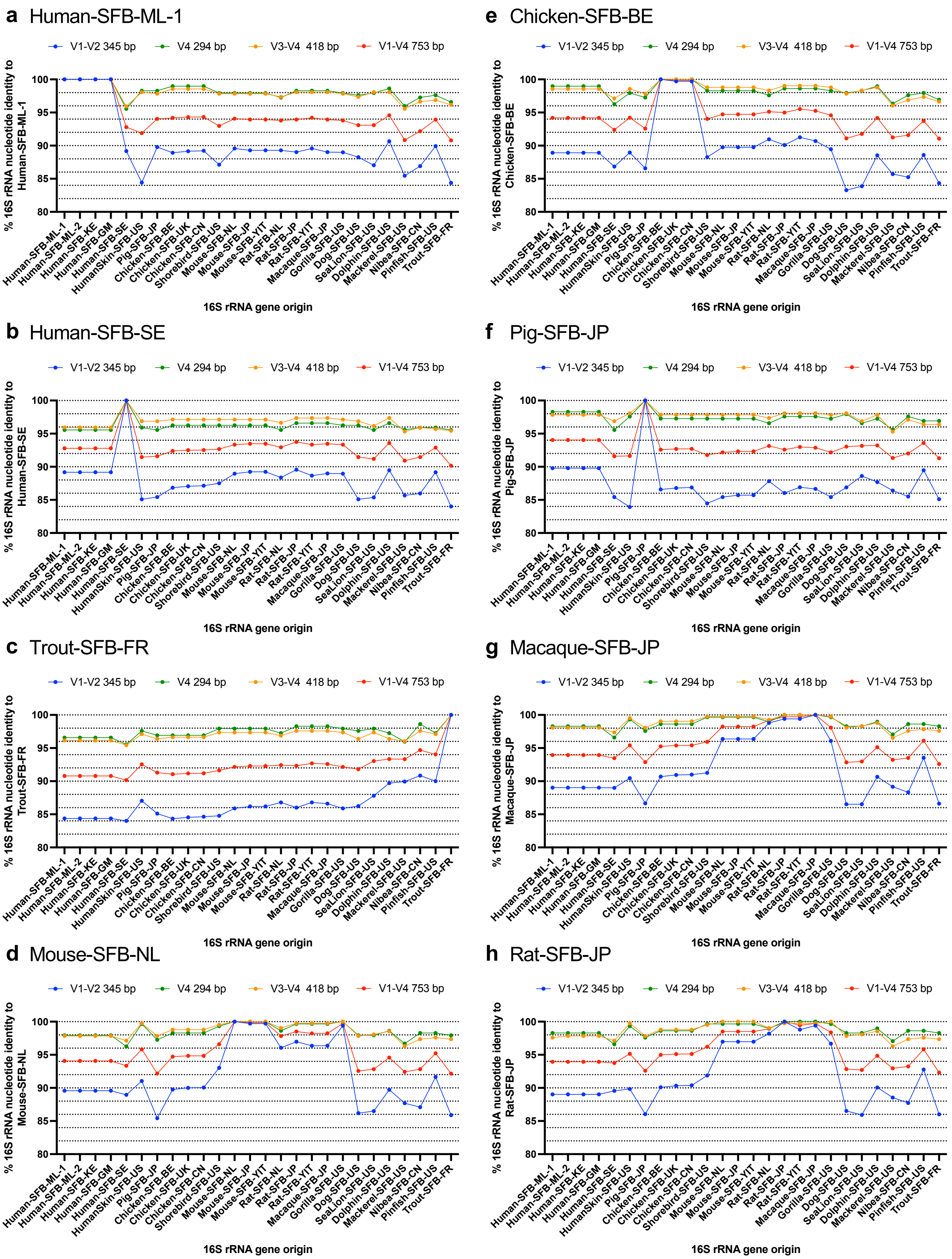

Supplementary Figure 6. Percent nucleotide identities of variable regions from selected reference SFB 16S rRNA gene sequences across SFB from various hosts. 16S rRNA gene sequence identity of the V1-V2, V4, V3-V4, and V1-V4 16S rRNA gene variable regions between SFB from various host and **a** Human-SFB-ML, **b** Human-SFB-SE, **c** Trout-SFB-FR, **d** Mouse-SFB-NL, **e** Chicken-SFB-BE, **f** Pig-SFB-JP, **g** Macaque-SFB-JP, and **h** Rat-SFB-JP.

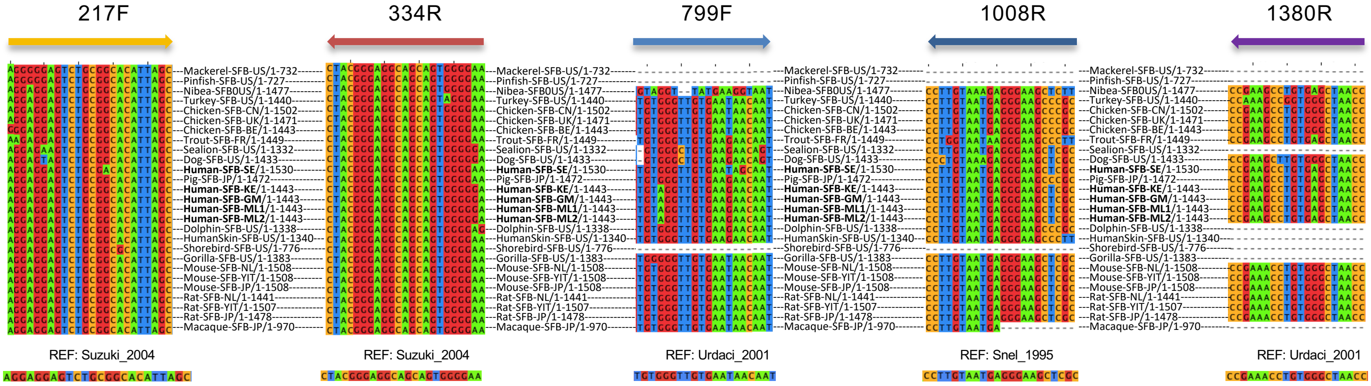

Supplementary Figure 7. Sequence variability in the 16S rRNA gene region for common primers to detect SFB in human samples. Highlight of alignment from Supplementary Fig. 3. Alignments of 16S regions of commonly used primer sequences for SFB detection across SFB from different host origins. The original non-human primer sequence and reference are included. Sequences were aligned with Clustal Omega and viewed using Jalview. The 16S rRNA gene sequence for Turkey-SFB-US (GCA_001655775.1) is derived from metagenomic data and is therefore not included in additional analyses. Nucleotides are color coded to increase clarity.

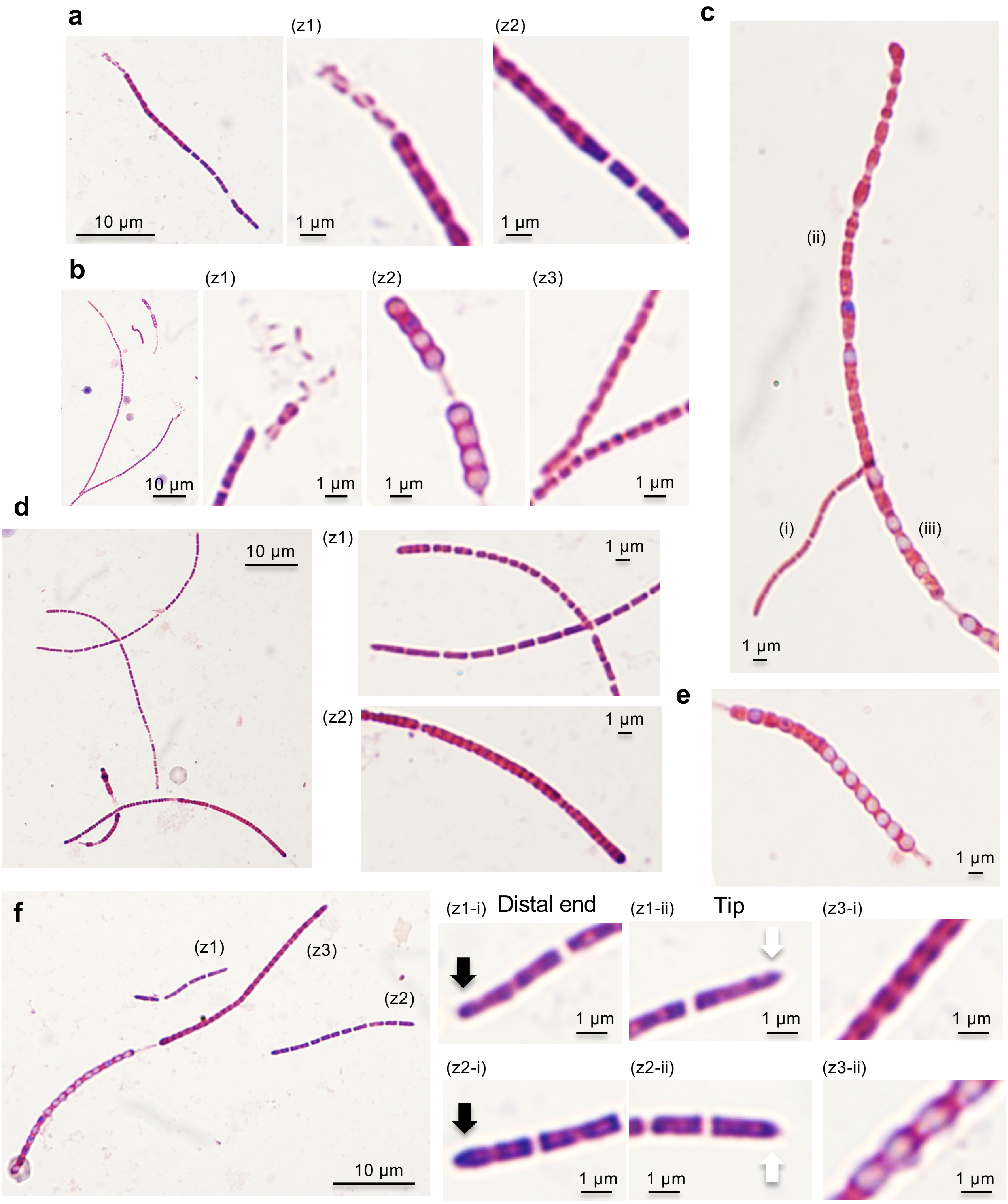

Supplementary Figure 8. Gram stains of SFB derived from intestinal contents of germfree mice monocolonized with Mouse-SFB-NL. Highlights of various SFB morphologies: **a** SFB filament with zooms to (z1) teardrop-shaped IOs being released from a bulbous filament end and (z2) a region of filament morphology transition. **b** SFB filaments with zooms to (z1) IOs near a filament remnant and filament tip, (z2) clustered spores within a disintegrating middle section, and (z3) sections of two filaments. **c** SFB filaments at varying stages including **c**(i) one with a smooth and thin morphology, and **c**(ii/iii) one with **c**(ii) bulbous sections and **c**(iii) sections with spores and a disintegrated region joining them. **d** SFB filaments with zooms to (z1) the end of two filaments showing a (at top) more bulbous and a (at bottom) smooth phenotype with defined septa, and (z2) a filament end showing a thicker and bulbous phenotype. **e** SFB filament remnant of a bulbous section including spores. **f** SFB filaments with zooms to (z1/z2) two filaments, to highlight the (i) distal end (black arrows) and (ii) tip (white arrows) of smooth and thin filaments, and **f**(z3) a third filament with a (i) bulbous and a (ii) spore-containing region.

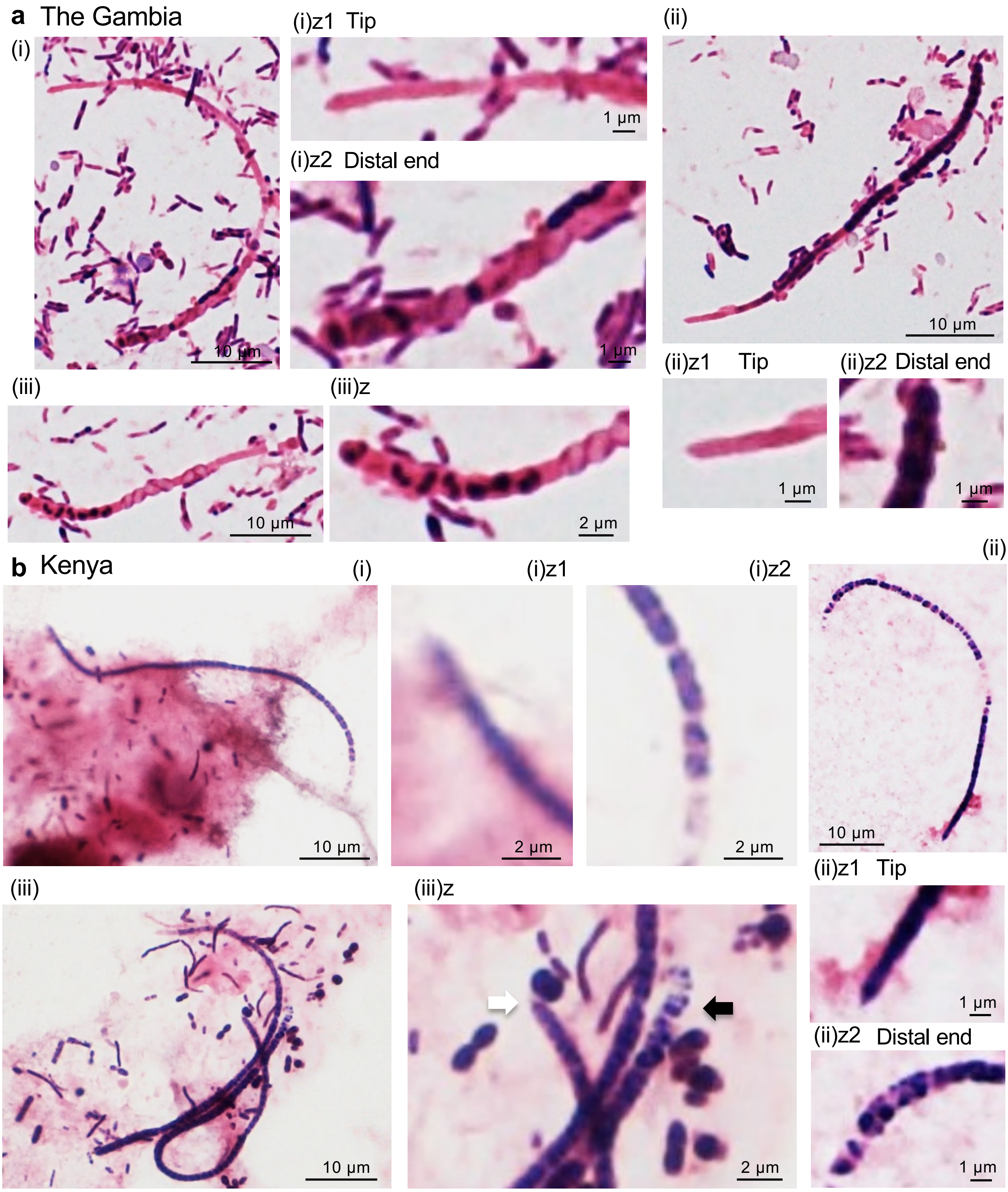

Supplementary Figure 9. Gram stains of fecal samples from The Gambia and Kenya including filaments with an SFB-like morphology. Gram stain of fecal samples from bioproject PRJNA234437. **a** The Gambia sample 102358 from a child without diarrhea. **a**(i/ii) A filament with a zoom to (z1) the thin end, including an apparently pointed tip, and to (z2) the bulbous distal end; **a**(iii) Filament with a smooth to bulbous transition and including a zoom (z) showing potential intracellular offsprings near the bulbous end as well as spores between the bulbous and thin smooth ends. **b** The Kenya sample 401080 from a child with non-dysenteric diarrhea. **b**(i) Filament with a zoom to (z1) the thin end and to (z2) the bulbous end showing filament disintegration; **b**(ii) Filament with a zoom to (z1) the thin end, including an apparent pointed tip, and to (z2) the bulbous distal end showing filament disintegration; **b**(iii) Two filaments with a zoom (z) highlighting the end with an apparent tip (white arrow) and the disintegrating distal end (black arrow) for one of the filaments.

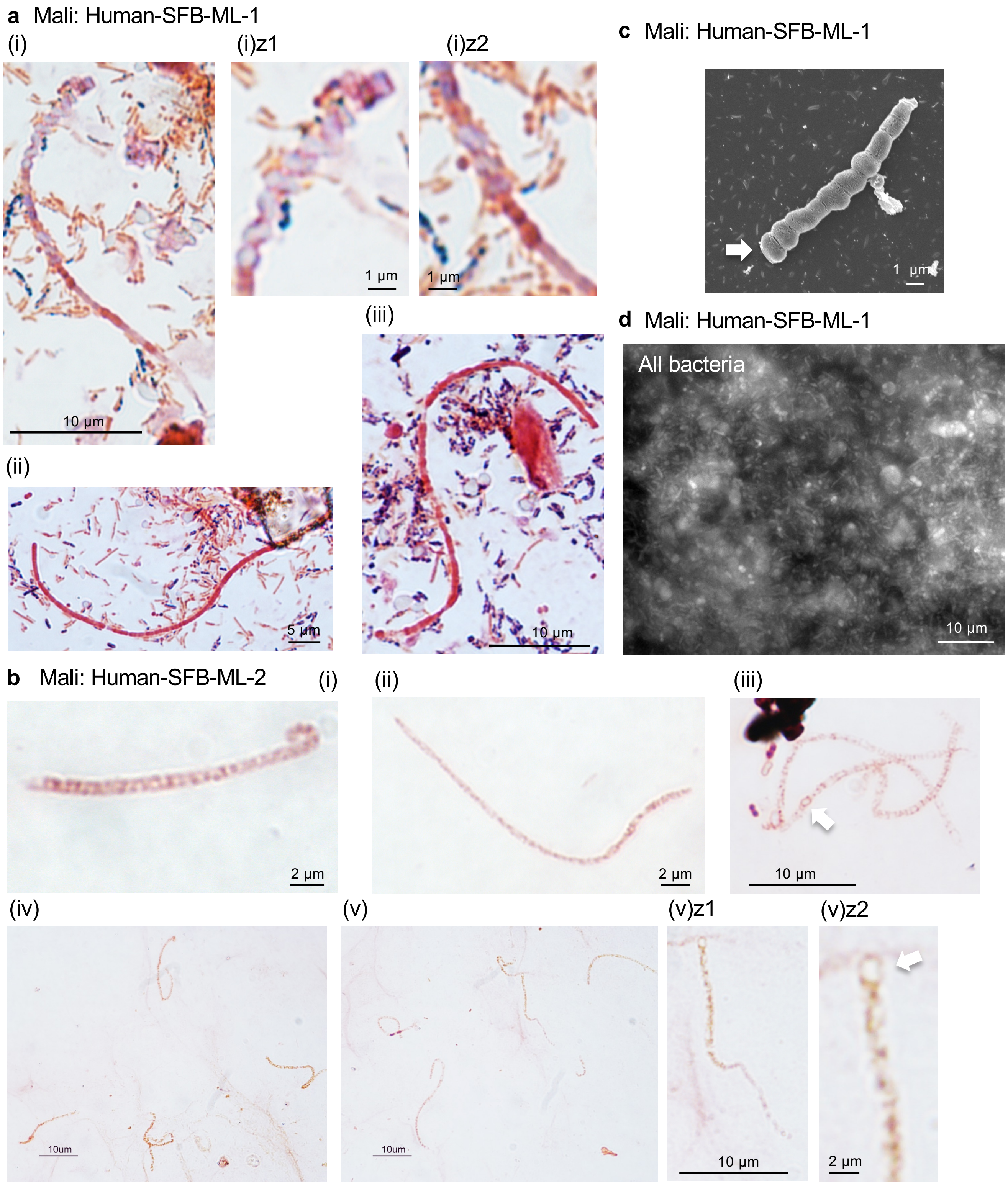

Supplementary Figure 10. Analysis of fecal samples from Mali from bioproject PRJNA234437. **a** Gram stains of the Mali sample 200340 (S340, Human-SFB-ML-1) from a child without diarrhea. **a**(i-iii) Filaments with an SFB-like morphology; **a**(i) Reproduction of image Fig. 1h(ii) with zooms to (z1) spores and (z2) a section of the filament with varying bulbous sections as well as spores; **a**(ii/iii) Filament with characteristic SFB bulbous sections. **b** Gram stains of the Mali sample 200195 (S195, Human-SFB-ML-2) from a child with non-dysenteric diarrhea including **b**(i-v) disintegrated filamentous bacteria and filaments with spores **b**(iii) and **b**(v) (z2), white arrows). **c** SEM image of a potentially broken (white arrow) bulbous SFB filament segment in the Mali S340 fecal sample. **d** All bacterial probe (Eub338) fluorescent *in situ* hybridization staining of the Mali S340 sample of Fig. 2b rendered in black and white to better visualize the dense bacterial aggregate.

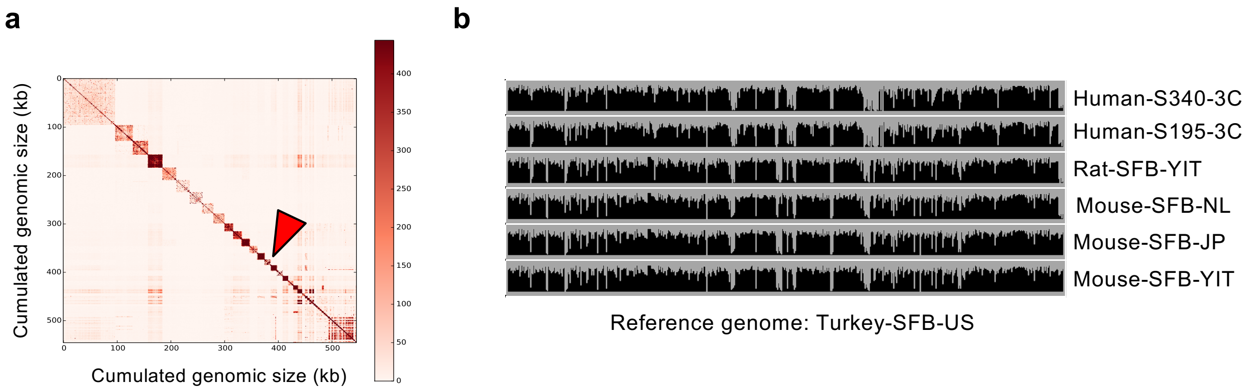

Supplementary Figure 11. Malian human draft SFB genome sequence analysis. a Chromosome conformation capture analysis raw contact map (1 vector = 100 kb) of the metagenome-assembled genomes (MAGs) obtained from the Human-SFB-ML-1 (S340) Mali fecal sample showing size-ordered MAGs (i.e. bins > 500 kb) with contact signal strength indicator. Red arrowhead highlights the SFB MAG (15th from top left). **b** Blast Score Ratio comparison of human Malian SFB draft genomes (S340-3C and S195-3C) to SFB genomes from various hosts with Turkey-SFB-US as the reference genome.

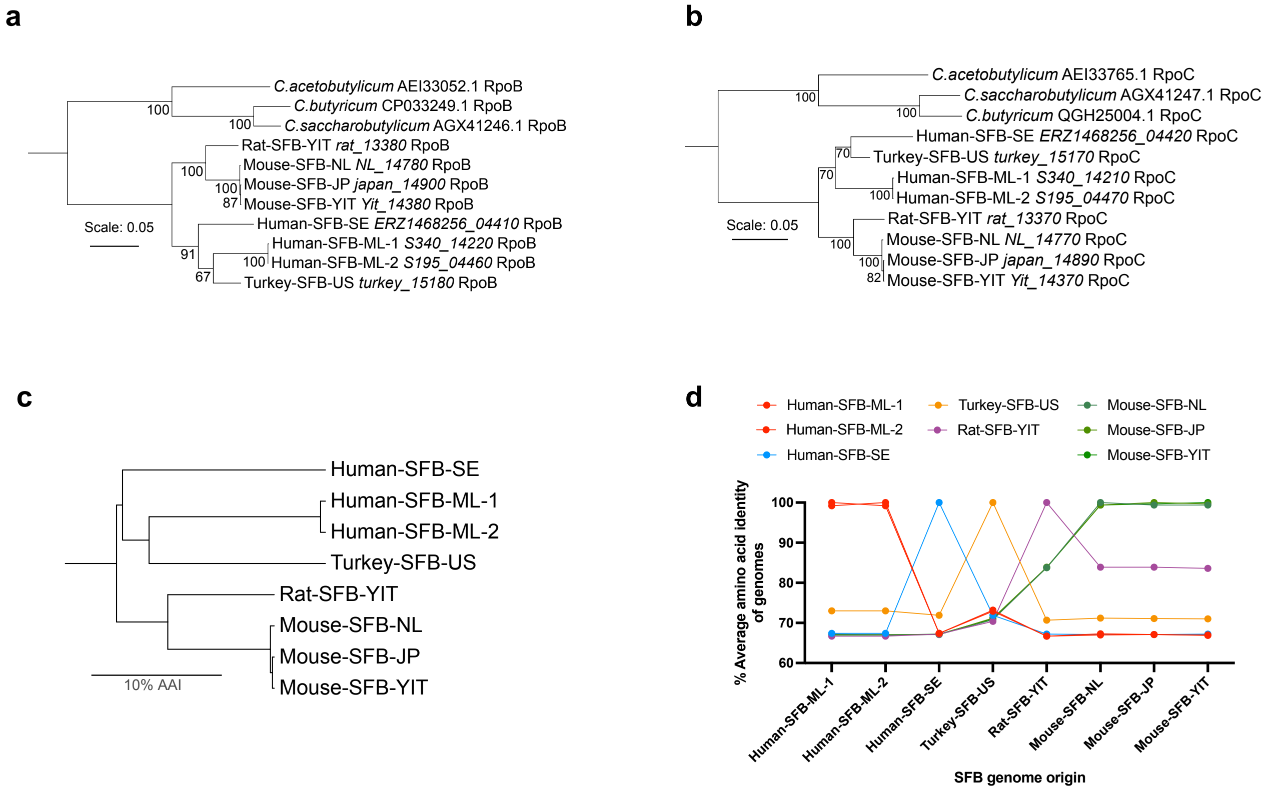

Supplementary Figure 12. Comparison of SFB from various hosts. Maximum likelihood phylogenetic tree of **a** RpoB and **b.** RpoC amino acid sequences across SFB from various hosts, including *Clostridium* outgroup species (italic). Proteins from SFB include their gene locus tag while proteins from *Clostridium* species include their NCBI reference number. **c** Dendrogram of the average amino acid sequence identity (AAI) between the whole genome proteome of SFB from various hosts. Scale indicates percent difference in AAI across SFB genomes. **d** Percent AAI plotted across SFB genomes from various hosts. **a/b** Phylogenetic trees include bootstrap values and the scale is amino acid substitutions per amino acid position.

a

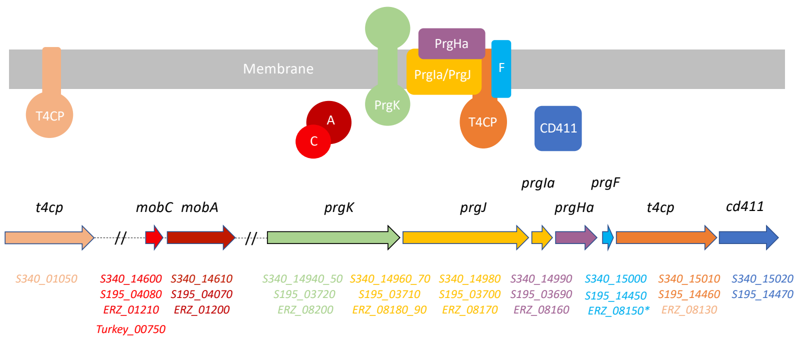

c

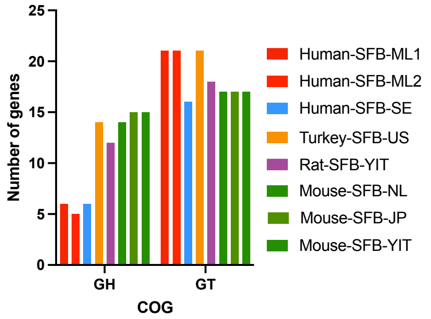

b

	S340_01050	S340_15010	S195_14460	ERZ1468256_08130
S340_01050_t4cp _TRAG family protein_GC_00001619	100,0	72,0	66,8	85,3
S340_15010_t4cp _TRAG family protein_GC_00002054	72,0	100,0	98,1	73,7
S195_14460_t4cp _TRAG family protein_GC_00002054	66,8	98,1	100,0	68,5
ERZ1468256_08130_t4cp _TRAG family protein_GC_00001619	85,3	73,7	68,5	100,0

	S340_14960	S340_14970	S195_03710	ERZ1468256_08180	ERZ1468256_08190
S340_14960 _FATA_*prgJ* _inner-membrane protein TraC_GC_00002100	100,0	0,0	99,5	51,4	0,0
S340_14970 _FATA_*prgJ* _Type IV secretory pathway, VirB4 components_GC_00001655	0,0	100,0	97,6	35,3	0,0
S195_03710 _FATA_*prgJ* _inner-membrane protein TraC_GC_00001655	99,5	97,6	100,0	44,9	53,5
ERZ1468256_08180 _FATA_*prgJ* _inner-membrane protein TraC_GC_00002100	51,4	35,3	44,9	100,0	0,0
ERZ1468256_08190 _FATA_*prgJ* _uncharacterized or unknown_GC_00001655	0,0	0,0	53,5	0,0	100,0

	S340_14600	S195_04080	ERZ1468256_01210	turkey_00750
S340_14600_mobC _Bacterial mobilisation protein MobC_GC_00002036	100,0	98,2	31,5	37,9
S195_04080_mobC _uncharacterized or unknown_GC_00002036	98,2	100,0	30,6	38,9
ERZ1468256_01210_mobC _uncharacterized or unknown_GC_00002113	31,5	30,6	100,0	72,6
turkey_00750_mobC _uncharacterized or unknown_GC_00002113	37,9	38,9	72,6	100,0

	S340_14610	S195_04070	ERZ1468256_01200
S340_14610_mobA _Relaxase/Mobilisation nuclease domain protein_GC_00002384	100,0	97,6	37,6
S195_04070_mobA _Relaxase/Mobilisation nuclease domain protein_GC_00002384	97,6	100,0	37,0
ERZ1468256_01200 _mobA _Relaxase/Mobilisation nuclease domain protein_GC_00002885	37,6	37,0	100,0

	S340_14940	S340_14950	S195_03720	ERZ1468256_08200
S340_14940_prgK _putative peptidase, M23 family_GC_00001605	100,0	0,0	98,5	37,7
S340_14950_prgK _uncharacterized or unknown_GC_00001605	0,0	100,0	99,1	0,0
S195_03720_prgK _putative peptidase, M23 family_GC_00001605	98,5	99,1	100,0	32,2
*ERZ1468256_08200_prgK_* uncharacterized or unknown_GC_00002792	38,3	0,0	28,2	100,0

	S340_14980	S195_03700	ERZ1468256_08170
S340_14980 _FATA_*prgla* _PrgI family protein_GC_00002150	100,0	97,0	43,2
S195_03700 _FATA_*prgla* _PrgI family protein_GC_00002150	97,0	100,0	41,4
ERZ1468256_08170 _FATA_*prgla* _PrgI family protein_GC_00002900	43,2	41,4	100,0

	S340_14990	S195_03690	ERZ1468256_08160
S340_14990 _FATA_*prgHa* _uncharacterized or unknown_GC_00002420	100,0	98,8	45,0
S195_03690 _FATA_*prgHa* _uncharacterized or unknown_GC_00002420	98,8	100,0	42,2
ERZ1468256_08160 _FATA_*prgHa* _uncharacterized or unknown_GC_00002584	45,0	42,2	100,0

	S340_15000	S195_14450	ERZ1468256_08150
S340_15000 _FATA_*prgF* _Maff2 family protein_GC_00001427	100,0	93,2	62,0
S195_14450 _FATA_*prgF_* Maff2 family protein_GC_00001427	93,2	100,0	68,2
ERZ1468256_08150 _FATA_*prgF_* Maff2 family protein_GC_00001427	62,0	68,2	100,0

	S340_15020	S195_14470
S340_15020 _FATA_*cd411* _uncharacterized or unknown_GC_00002396	100,0	98,1
S195_14470 _FATA_*cd411* _uncharacterized or unknown_GC_00002396	98,1	100,0

d

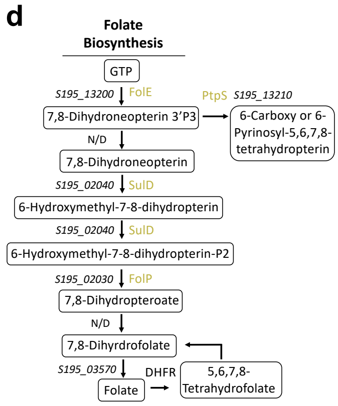

e

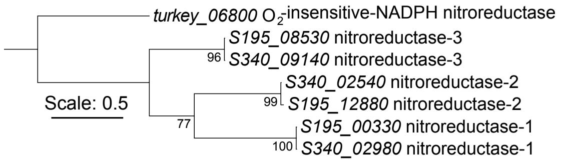

Supplementary Figure 13. Comparison of select genome features of SFB from various hosts. a Schematic of the components of the putative conjugation system identified in human SFB genomes from Mali (S340 and S195) and Sweden (ERZ). Included are the reference genes of the genomes. Gene references, gene sequence schematics and the corresponding protein schematics are color coded for clarity. **b** Sequence identity comparison of components of the putative conjugation system of human SFB. Percent amino acid identity across human SFB genomes from Mali (S195 and S340) and Sweden (ERZ1468256), and the Turkey-SFB-US genome. Annotations include the locus tag for the gene (italic) and corresponding protein (non-italic), gene name (when available), protein annotation and gene cluster number. **c** The number of glycosyl hydrolases (GH) and glycosyl transferases (GT) genes in the SFB genomes form various hosts. COG: Cluster of Orthologous Genes. **d** Folate biosynthetic pathways with enzymes predicted to be present in all SFB genomes in black and those only present in the Human-SFB-ML and Turkey-SFB-US genomes highlighted in yellow-green. Includes the reference gene locus tags of Human-SFB-ML-2. Substrates and products are boxed. DHFR: Dihydrofolate reductase N/D: not determined. **e** Maximum likelihood phylogenetic tree of nitroreductases absent in the closed SFB genomes of mouse and rat SFB but present in the genomes of Human-SFB-ML and Turkey-SFB-US. Scale: amino acid substitutions per amino acid position. Nitroreductase description includes the gene locus tag.

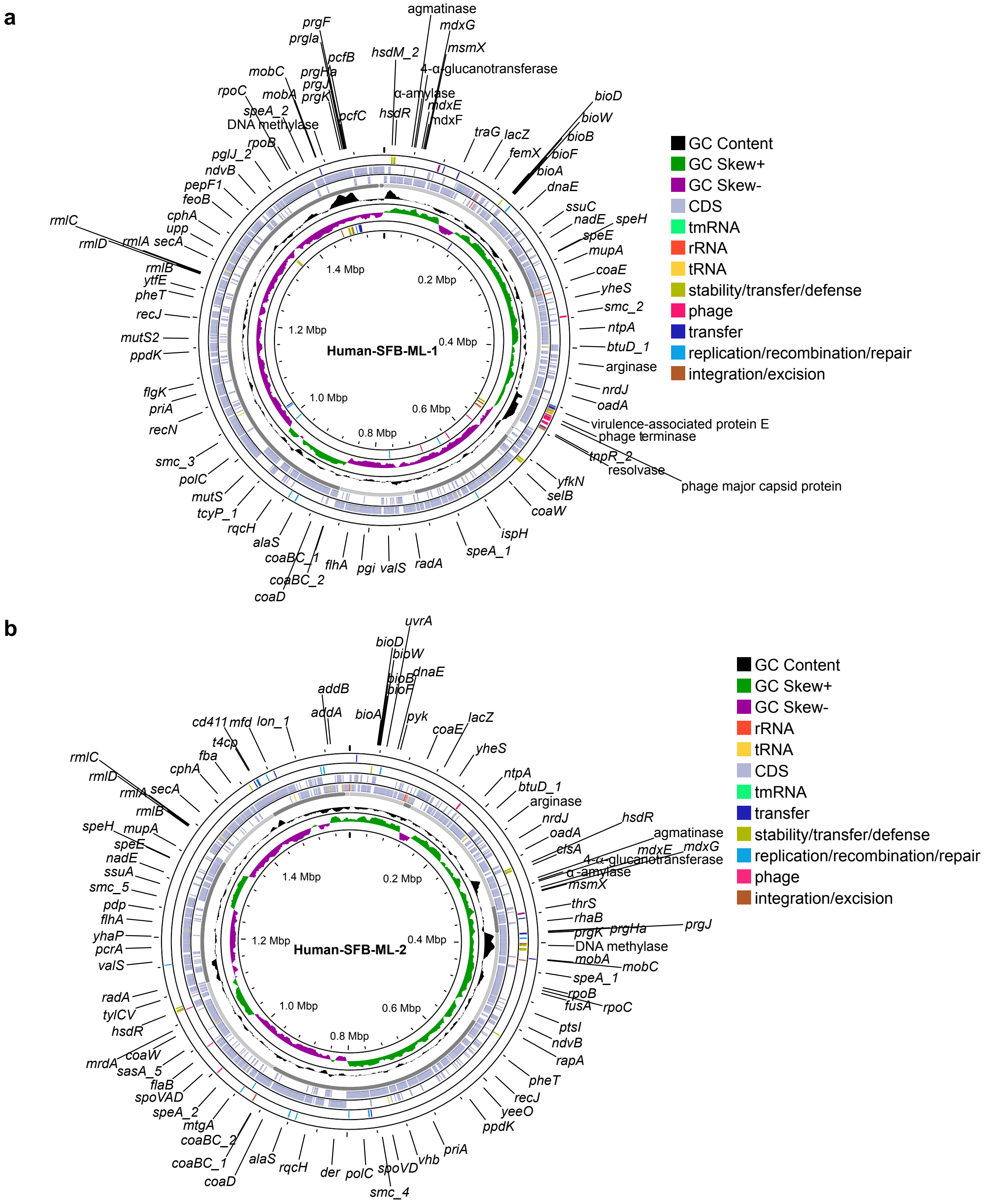

Supplementary Figure 14. Genome features of human SFB from Malian origin. SFB genome sequence from the **a** Human-SFB-ML-1 (S340) and **b** Human-SFB-ML-2 (S195) Malian fecal samples showing various genomic features including GC content and selected predicted genes with color highlighting of genes in the defense and mobile element categories. High GC content regions include those harboring the hsdR restriction methylation system, the putative conjugation system of the FATA subtype found in both genomes, and a phage island in Human-SFB-ML-1. Genomic regions harboring the biotin, coenzyme A and rhamnose synthesis genes as well as the glycogen and starch utilization island are indicated and lie outside the high GC content regions. For clarity, for some genes, gene symbols were replaced with a more descriptive labeling to highlight predicted enzymatic activity or function.

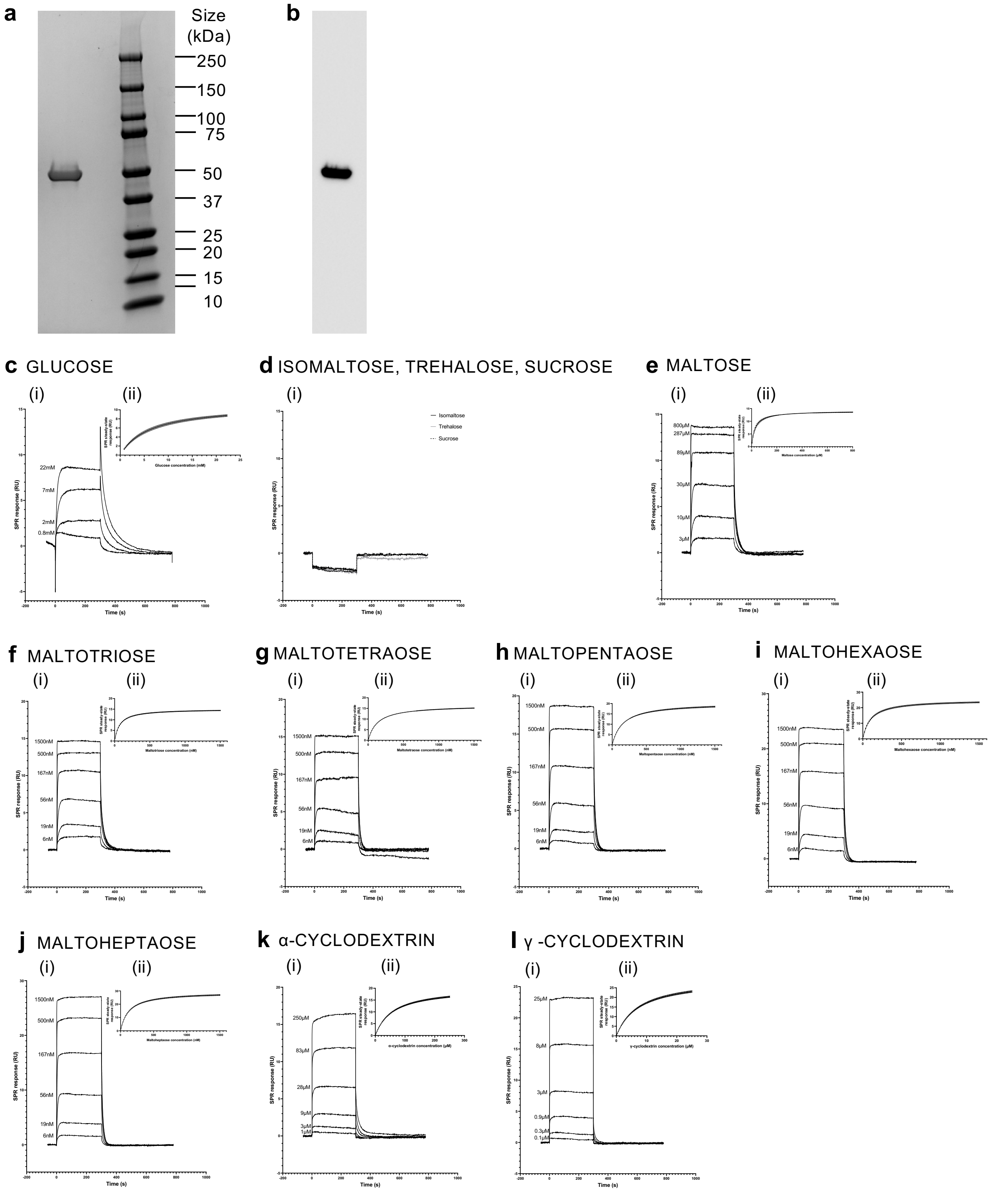

Supplementary Figure 15. Expression and characterization of MdxE from Human-SFB-ML. a/b Purification of recombinant His-tagged MdxE. **a** SDS-PAGE gel stained with Coomassie brilliant blue R-250 showing the purified recombinant MdxE protein and a molecular weight marker; **b** Western blot of purified recombinant MdxE protein using an anti-polyhistidine antibody (1:5000 dilution). **c-l.** Surface Plasmon Resonance (SPR) of the MdxE-sugar interaction. **c-l** (i) Sensorgrams obtained for the MdxE-sugar interaction and **c**,**e-l** (ii) the steady-state affinity fitting to calculate MdxE-sugar binding affinity (KD) for **c** glucose; **d** isomaltose, trehalose and sucrose; **e** maltose; **f** maltotriose; **g** maltotetraose; **h** maltopentaose; **i** maltohexaose; **j** maltoheptaose; **k** α-cyclodextrin; and **l** γ-cyclodextrin. For (**d**) isomaltose, trehalose and sucrose, no steady affinity fitting could be performed due to a lack of binding. SPR responses are given in resonance units (RU) where one RU corresponds to the accumulation of 1pg/mm^2 of sugar after the subtraction of the response values obtained in an empty flow cell. Sugar concentrations tested are indicated next to each sensorgram.

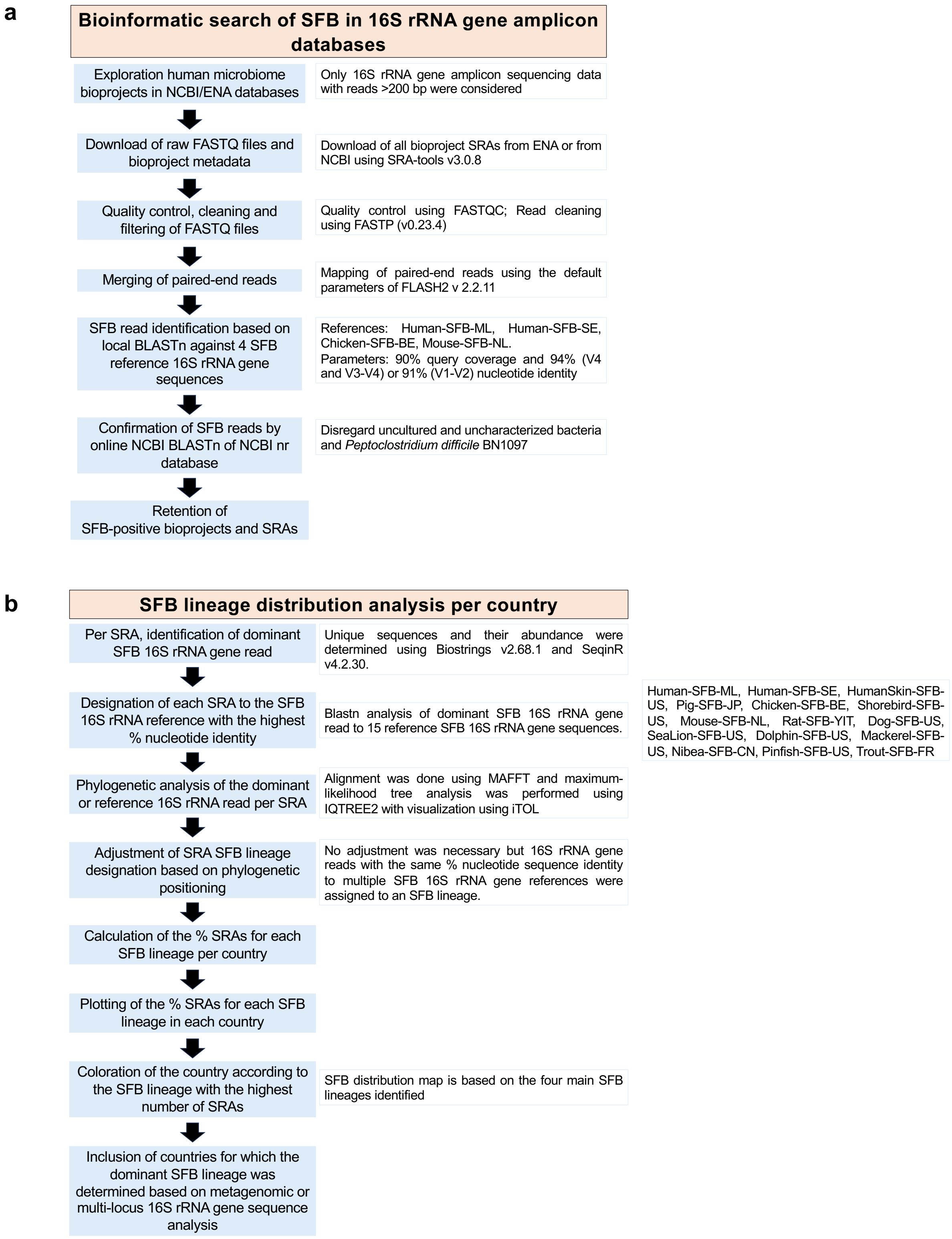

Supplementary Figure 16. Flowchart of the bioinformatic analysis performed to identify SFB-positive 16S rRNA gene reads in publicly available datasets. a Pipeline for the bioinformatic search of SFB-positive bioprojects. **b** Pipeline for the SFB lineage distribution per country.

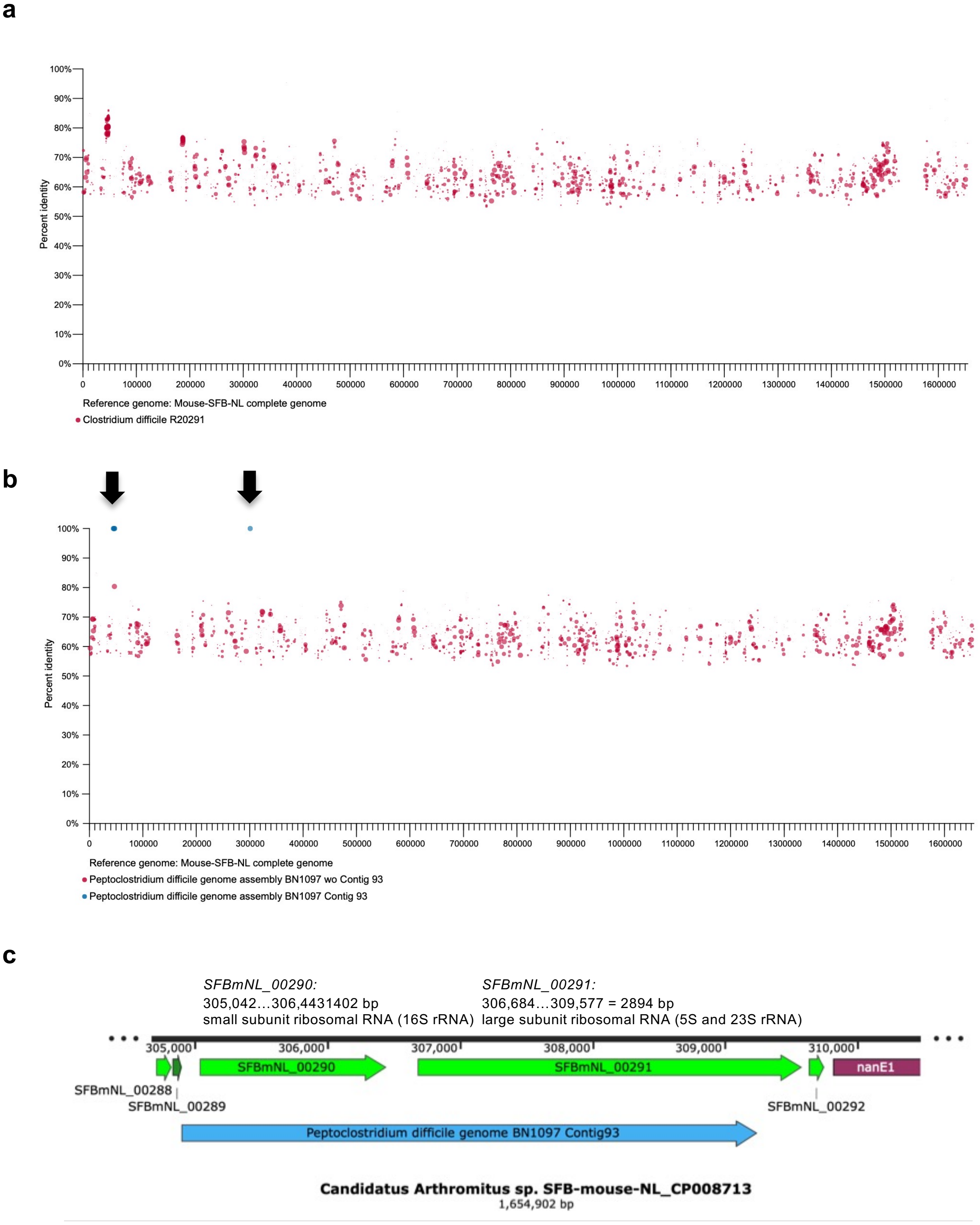

Supplementary Figure 17. *Peptoclostridium difficile* BN1097 genome contig analysis identifies a wrongly incorporated rRNA gene sequence island. Comparison, using Simiplot with a minimum window size of 4000 bp, of the Mouse-SFB-NL genome (CP008713) to **a** the closed *C. difficile* genome (R2021) or **b** the *P. difficile* contigs comprising the genome BN1097 with a separate analysis (blue) for contig93. Black arrows highlight unusual high nucleotide identity in (**b**) not seen in (**a**). **c** Schematic of the genomic region of Mouse-SFB-NL containing the 5S, 23S and 16S rRNA genes covered at a 100% nucleotide identity by contig93 of the *P. difficile* genome BN1097.

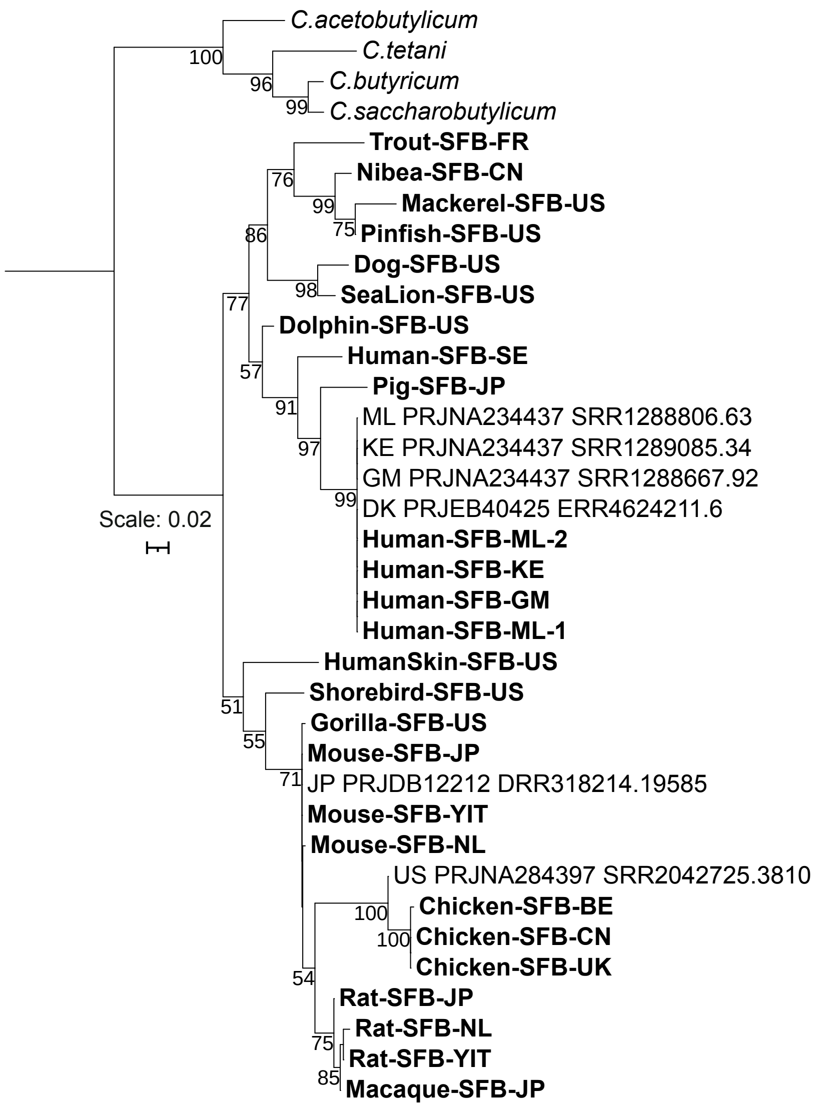

Supplementary Figure 18. Maximum likelihood phylogenetic tree of the dominant SFB 16S rRNA gene read for bioprojects of the V1-V2 16S rRNA gene sequence region. Maximum likelihood phylogenetic trees with *Clostridium* outgroups in italic and reference 16S rRNA gene sequences are in bold. Bioproject 16S rRNA gene reads are labeled with the two-letter country code of the country of origin, bioproject number, SRA number, and sequence read number. The tree includes bootstrap values and the scale is nucleotide substitutions per nucleotide position.

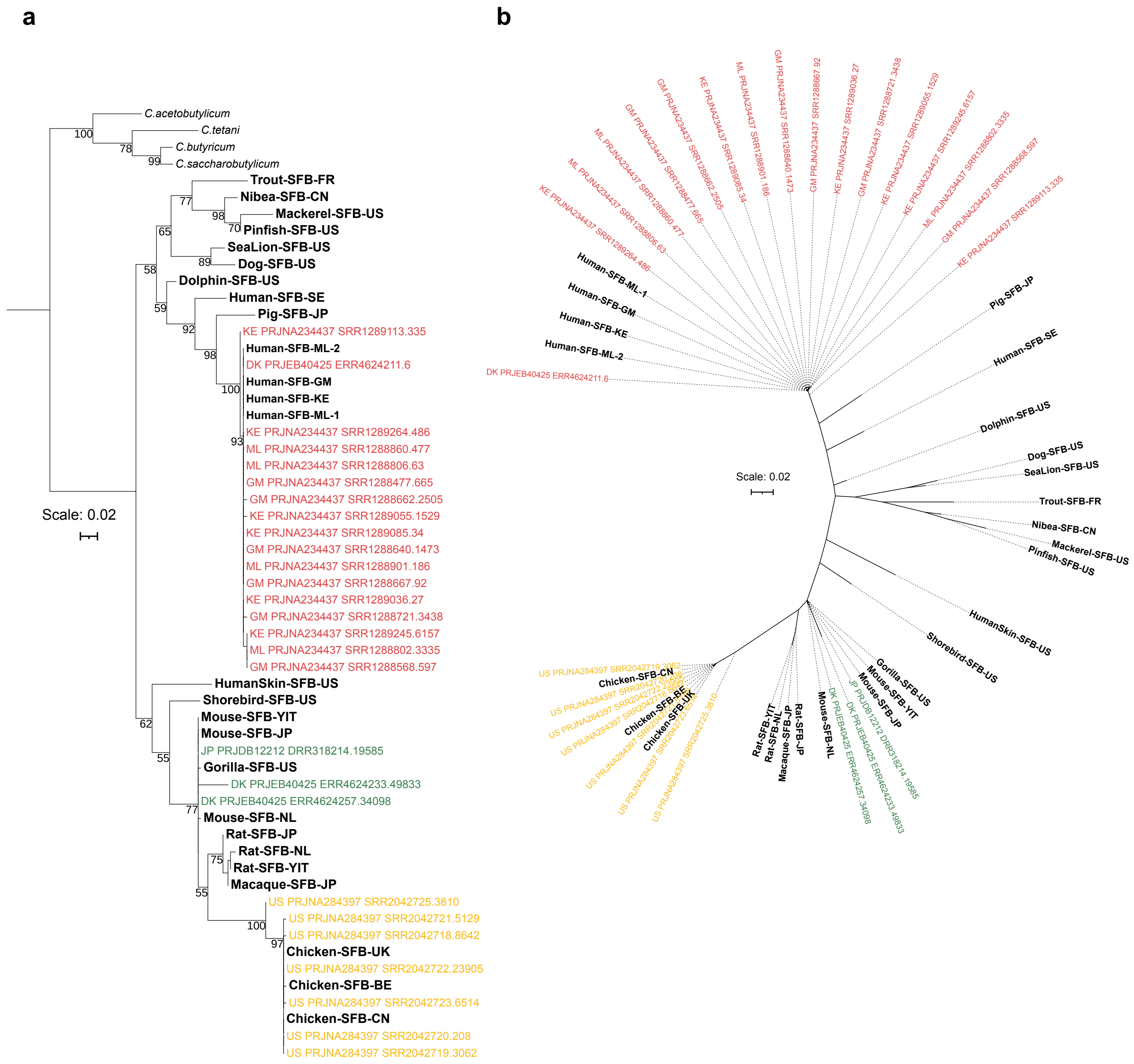

Supplementary Figure 19. Maximum likelihood phylogenetic trees of the dominant SFB 16S rRNA gene read for SRAs from bioprojects of the V1-V2 16S rRNA gene region. a/b Maximum likelihood phylogenetic trees **a** with and **b** without *Clostridium* outgroups (italic). The dominant 16S rRNA gene read per SRA is labeled with the two-letter country code of the country of origin, bioproject number, SRA number and sequence read number. SFB reference 16S rRNA gene sequences are in bold and SRAs are colored based on the SFB reference 16S rRNA gene sequences they are closest to (red: Human-SFB-ML, green: mouse SFB, yellow: chicken SFB). **a** Tree includes bootstrap values and for (**a/b**) the scale is nucleotide substitutions per nucleotide position.

a 16S rRNA gene V4 region

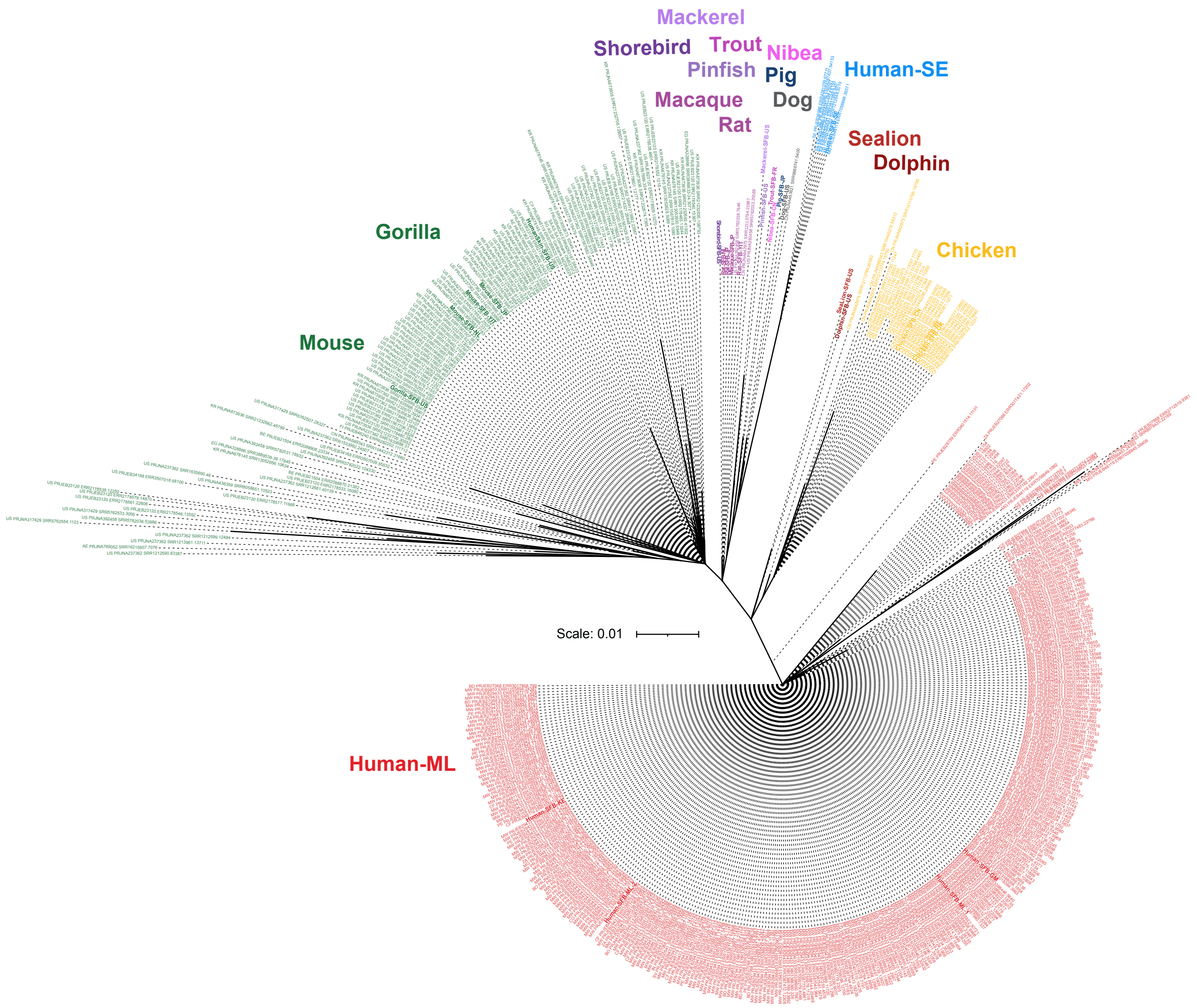

b 16S rRNA gene V3-V4 region

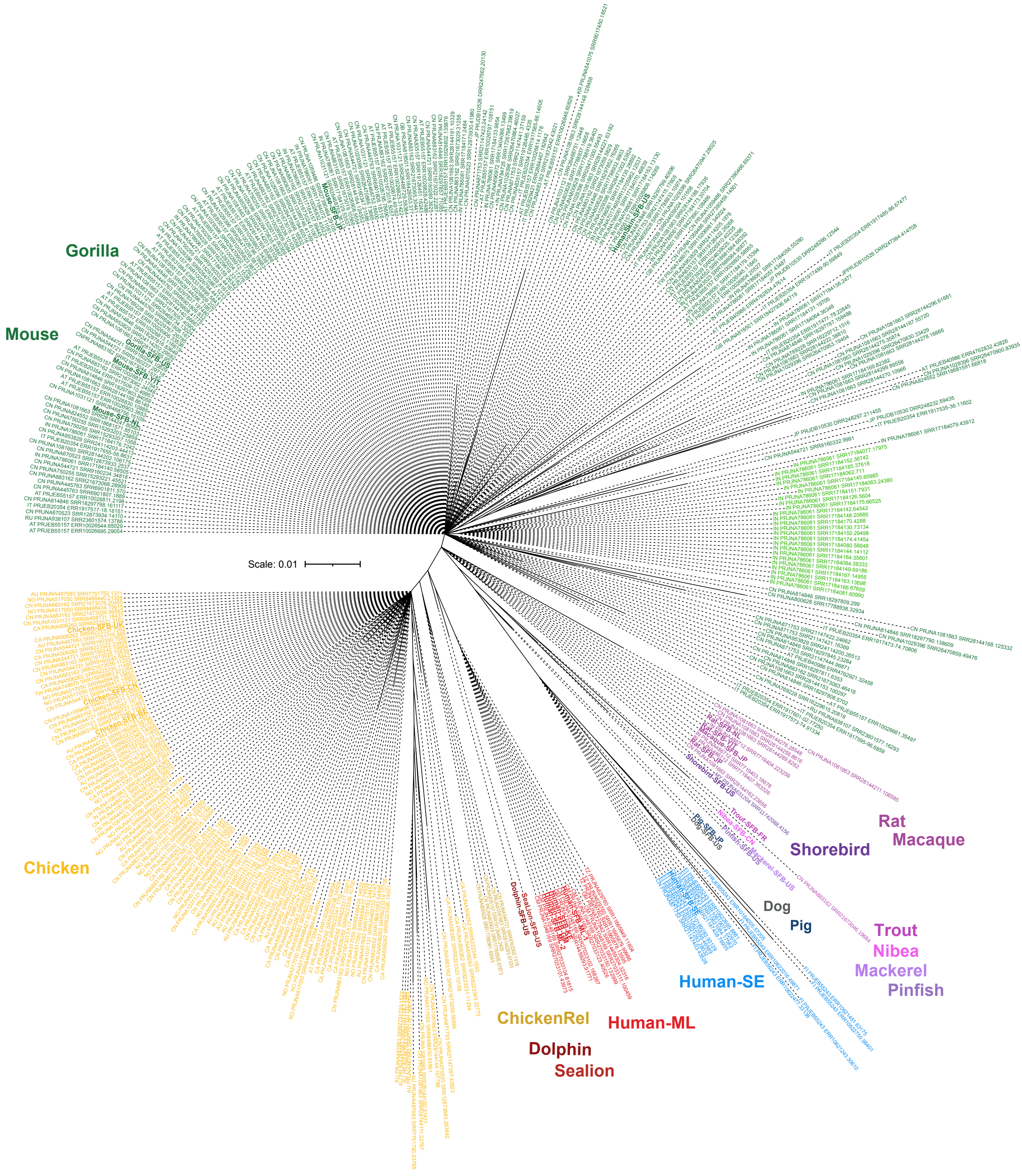

Supplementary Figure 20. Maximum likelihood phylogenetic trees of the dominant SFB 16S rRNA gene read for all SRAs from bioprojects of the V4 and V3-V4 16S rRNA gene region. Unrooted maximum likelihood phylogenetic trees of the **a** V4 and **b** V3-V4 16S rRNA gene region without *Clostridium* outgroups. The dominant 16S rRNA gene read per SRA is labeled with the two-letter country code of the country of origin, bioproject number, the SRA number, and the sequence read number. SRAs are colored based on the SFB reference 16S rRNA gene sequences they are closest to. The scale is in nucleotide substitutions per nucleotide position.

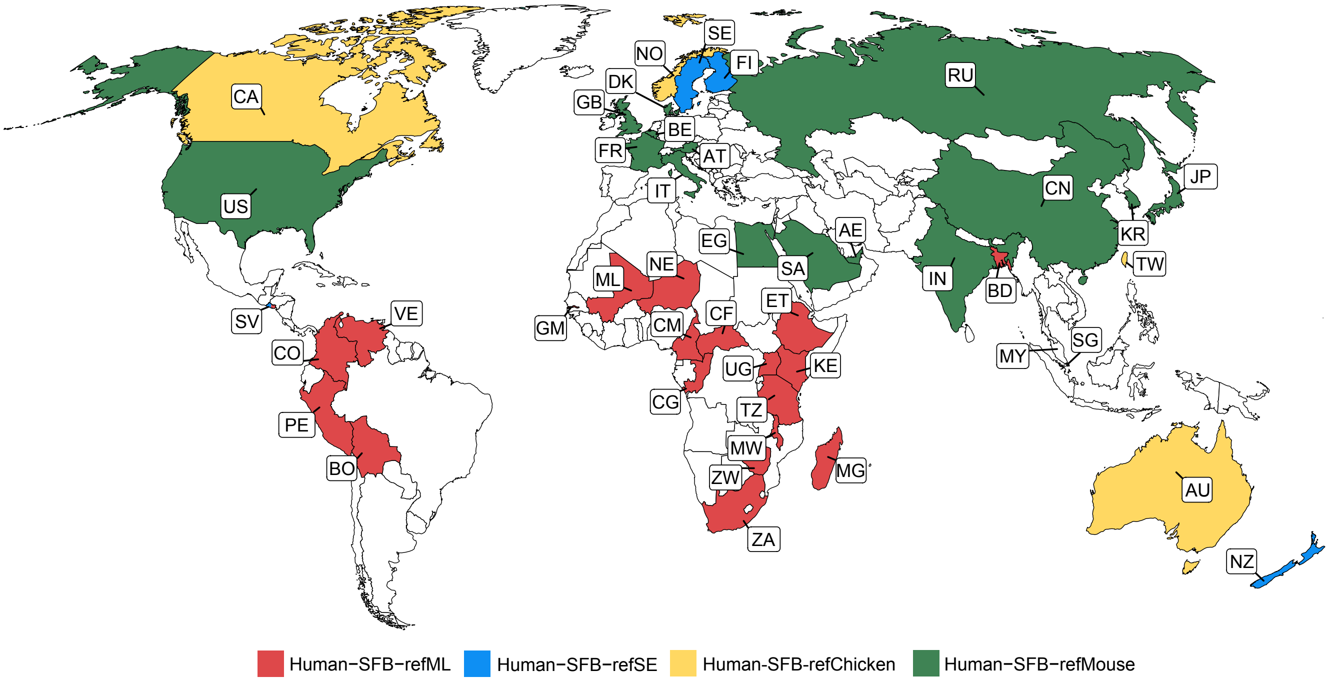

Supplementary Figure 21. Global Human SFB identification and lineage distribution. World map highlighting countries according to the most abundant SFB lineage identified in the 16S rRNA gene and metagenomic samples analyzed for each country (Supplementary Data 7). Human SFB lineage designation is based on the dominant SFB reference 16S rRNA gene sequence most prevalent in the SRA-based analysis of each country. Countries are indicated using their two-letter country code. AE: Arab Emirates, AT: Austria, AU: Australia, BD: Bangladesh, BE: Belgium, BO: Bolivia, CA: Canada, CF: Central African Republic, CG: Republic of the Congo, CM: Cameroon, CN: China, CO: Columbia, DK: Denmark, EG: Egypt, ET: Ethiopia, FI: Finland, FR: France, GB: Great Britain, GM: The Gambia, IN: India, IT: Italy, JP: Japan, KE: Kenya, KR: South Korea, MG: Madagascar, ML: Mali, MW: Malawi, MY: Malaysia, NE: Niger, NO: Norway, NZ: New Zealand, PE: Peru, RU: Russia, SA: Saudi Arabia, SE: Sweden, SG: Singapore, SV: El Salvador, TW: Taiwan, TZ: Tanzania, UG: Uganda, US: United States of America, VE: Venezuela, ZA: South Africa, ZW: Zimbabwe.

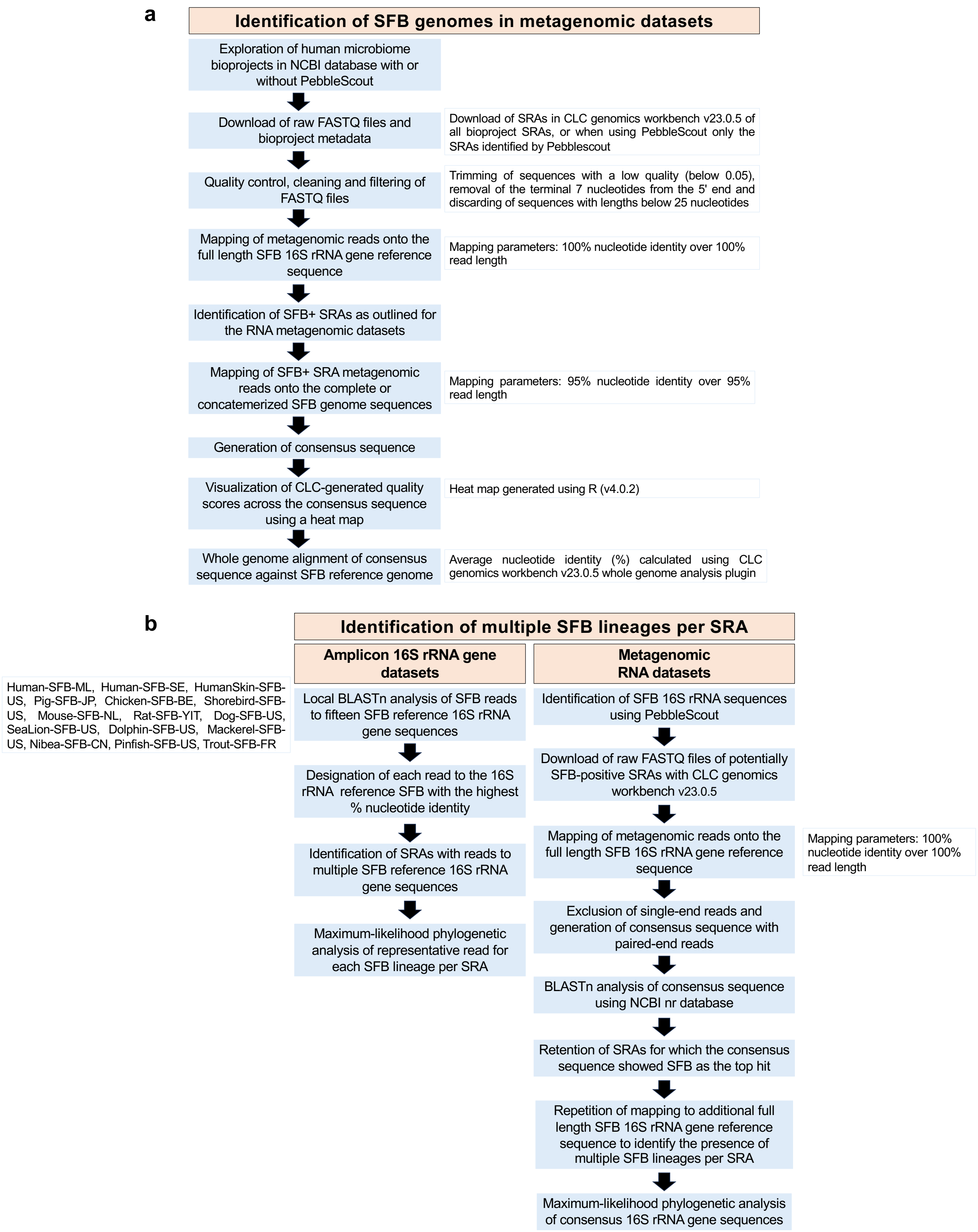

Supplementary Figure 22. Flowchart of the bioinformatic identification of SFB genomes in metagenomic datasets and co-occurrence of multiple SFB lineages within a single sample. a Outline for the identification of SFB genome identification in metagenomic datasets. **b** Outline for the identification of multiple SFB lineages in individual SRAs based on percent nucleotide identity to SFB 16S rRNA gene reference sequences as well as phylogenetic confirmation.

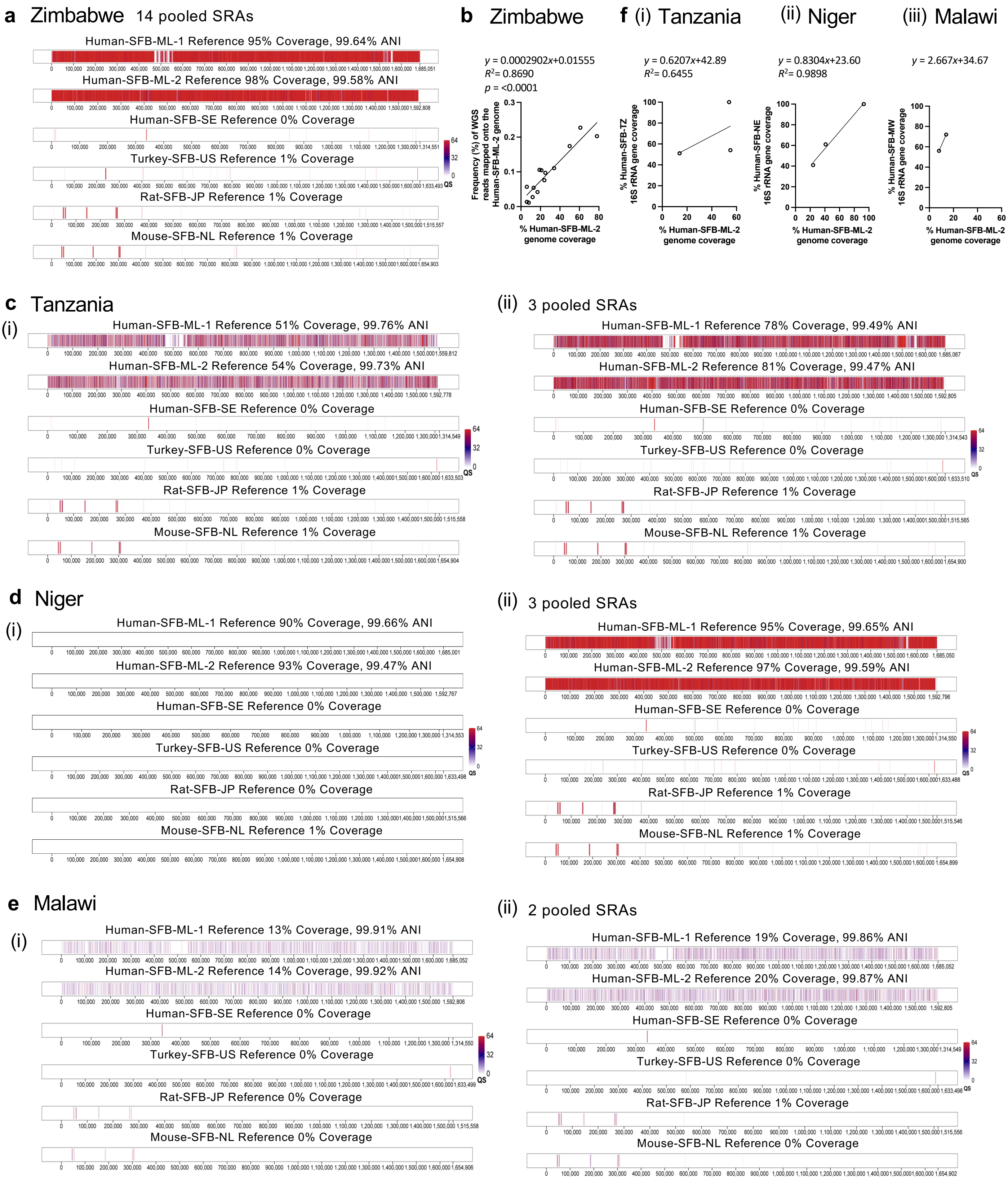

a Zimbabwe 14 pooled SRAs
Human-SFB-ML-1 Reference 95% Coverage, 99.64% ANI
Human-SFB-ML-2 Reference 98% Coverage, 99.58% ANI
Human-SFB-SE Reference 0% Coverage
Turkey-SFB-US Reference 1% Coverage
Rat-SFB-JP Reference 1% Coverage
Mouse-SFB-NL Reference 1% Coverage
b Zimbabwe
y = 0.0002902x+0.01555
R2= 0.8690
p = <0.0001
Frequency (%) of WGS reads mapped onto the Human-SFB-ML-2 genome
% Human-SFB-ML-2 genome coverage
f (i) Tanzania
y = 0.6207x+42.89
R2= 0.6455
% Human-SFB-TZ 16S rRNA gene coverage
(ii) Niger
y = 0.8304x+23.60
R2= 0.9898
% Human-SFB-NE 16S rRNA gene coverage
(iii) Malawi
y = 2.667x+34.67
% Human-SFB-MW 16S rRNA gene coverage
c Tanzania
(i)
Human-SFB-ML-1 Reference 51% Coverage, 99.76% ANI
Human-SFB-ML-2 Reference 54% Coverage, 99.73% ANI
Human-SFB-SE Reference 0% Coverage
Turkey-SFB-US Reference 0% Coverage
Rat-SFB-JP Reference 1% Coverage
Mouse-SFB-NL Reference 1% Coverage
(ii) 3 pooled SRAs
Human-SFB-ML-1 Reference 78% Coverage, 99.49% ANI
Human-SFB-ML-2 Reference 81% Coverage, 99.47% ANI
Human-SFB-SE Reference 0% Coverage
Turkey-SFB-US Reference 0% Coverage
Rat-SFB-JP Reference 1% Coverage
Mouse-SFB-NL Reference 1% Coverage
d Niger
(i)
Human-SFB-ML-1 Reference 90% Coverage, 99.66% ANI
Human-SFB-ML-2 Reference 93% Coverage, 99.47% ANI
Human-SFB-SE Reference 0% Coverage
Turkey-SFB-US Reference 0% Coverage
Rat-SFB-JP Reference 0% Coverage
Mouse-SFB-NL Reference 1% Coverage
(ii) 3 pooled SRAs
Human-SFB-ML-1 Reference 95% Coverage, 99.65% ANI
Human-SFB-ML-2 Reference 97% Coverage, 99.59% ANI
Human-SFB-SE Reference 0% Coverage
Turkey-SFB-US Reference 0% Coverage
Rat-SFB-JP Reference 1% Coverage
Mouse-SFB-NL Reference 1% Coverage
e Malawi
(i)
Human-SFB-ML-1 Reference 13% Coverage, 99.91% ANI
Human-SFB-ML-2 Reference 14% Coverage, 99.92% ANI
Human-SFB-SE Reference 0% Coverage
Turkey-SFB-US Reference 0% Coverage
Rat-SFB-JP Reference 0% Coverage
Mouse-SFB-NL Reference 0% Coverage
(ii) 2 pooled SRAs
Human-SFB-ML-1 Reference 19% Coverage, 99.86% ANI
Human-SFB-ML-2 Reference 20% Coverage, 99.87% ANI
Human-SFB-SE Reference 0% Coverage
Turkey-SFB-US Reference 0% Coverage
Rat-SFB-JP Reference 1% Coverage
Mouse-SFB-NL Reference 0% Coverage
QS

Supplementary Figure 23. SFB identification in metagenomic data from bioprojects originating in Tanzania, Niger, and Malawi. **a** Mapping of trimmed sequence reads from the Zimbabwe bioproject PRJEB51728 with fourteen SFB-positive SRAs to various SFB genomes. **b** Correlation between the frequency of mapped reads from PRJEB51728 SRAs and the coverage of the Human-SFB-ML-2 genome. **c** Mapping of trimmed sequence reads from the Tanzania bioproject PRJEB49206 **c**(i) SRA ERR7746794 from a 6 month-old infant of the Hadza hunter-gatherer tribe or **c**(ii) all three SFB-positive SRAs to various SFB genomes. **d** Mapping of trimmed sequence reads from the Niger bioproject PRJNA739008 **d**(i) SRA SRR14860444 of a child or **d**(ii) all three SFB-positive SRAs to various genomes. **e** Mapping of trimmed sequence reads from the Malawi bioproject PRJEB24771 **e**(i) SRA ERR2368082 or **e**(ii) together with SRA ERR2367738, derived from two 8month-old infants to SFB genomes of various hosts. **f** Correlation between SFB 16S rRNA gene sequence coverage and the coverage of the Human-SFB-ML-2 genome through mapping of trimmed sequence reads of SFB-positive SRAs from **f**(i) Tanzania bioproject PRJEB49206, **f**(ii) Niger bioproject PRJNA739008, and **f**(iii) Malawi bioproject PRJEB24771. Mapping of cleaned and trimmed reads was performed at 95% and 100% mapping stringency for nucleotide identity and read length for SFB genomes and 16S rRNA gene sequences, respectively, using the CLC genomic software package. QS: Quality score.

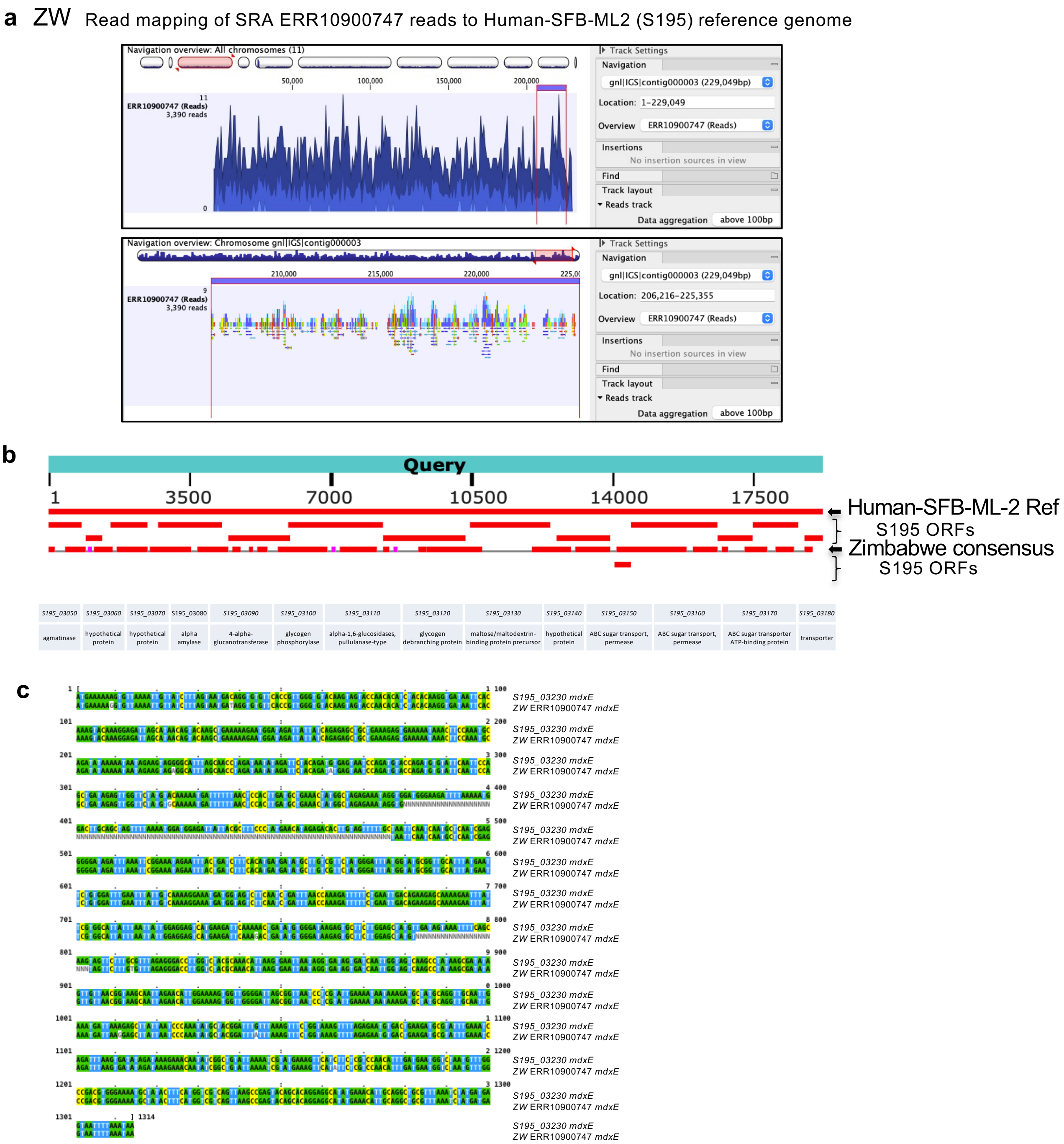

Supplementary Figure 24. Identification of the glycogen utilization island in metagenomic data in a human fecal sample of Zimbabwean origin. Metagenomic sequence reads with a minimum 95% sequence length and sequence identity from the SRA ERR10900747 of the Zimbabwe (ZW) bioproject PRJEB51728 were mapped onto the Human-SFB-ML-2 genome using CLC Genomics Workbench software. **a** Highlight of the coverage map of Human-SFB-ML-2 contig 3 containing the zoomed-in maltooligosaccharide locus. **b** Clustal Omega alignment, viewed with MView, of the genomic region of Human-SFB-ML-2 covering the maltoligosaccharide module-containing locus with its predicted CDS and the Zimbabwe SRA ERR10900747 consensus sequence. **c** Alignment of the *mdxE* gene of Human-SFB-ML-2 and the metagenomic read consensus of SRA ERR10900747 for the *mdxE* gene using read mapping parameters of a minimum of 99% sequence length and 98% sequence identity.

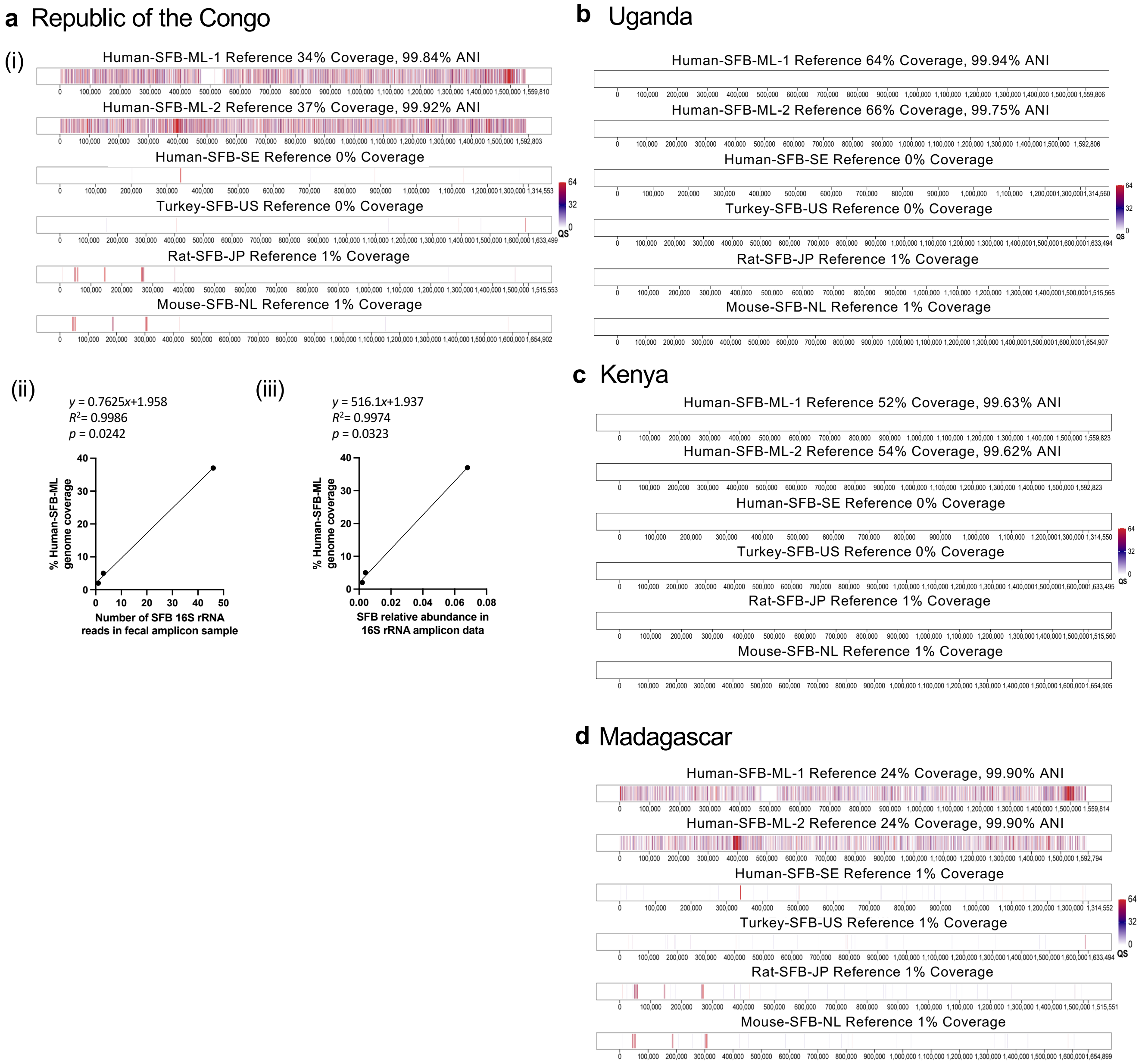

Supplementary Figure 25. SFB identification in metagenomic data from bioprojects originating in the Congo, Uganda, Kenya, and Madagascar. a Mapping of trimmed sequence reads from the Republic of the Congo bioproject PRJNA539933 **a**(i) SRA SRR8978278 to SFB genomes of various hosts and **a**(ii/iii) the correlation between SFB **a**(ii) 16S rRNA gene read number or **a**(iii) relative abundance and the percent Human-SFB-ML genome coverage obtained with 5-6.4 million metagenomic reads per sample for the three SFB-positive SRAs from the 16S rRNA amplicon-based SRAs of the same bioproject. **b** Mapping of trimmed sequence reads of the Uganda bioproject PRJNA1137832 SRA SRR29888954 from a child's fecal sample to SFB genomes of various hosts. **c** Mapping of trimmed sequence reads of the Kenya bioproject PRJNA1045584 SRA SRR27430646 from an infant fecal sample to SFB genomes of various hosts. **d** Mapping of trimmed sequence reads of the Madagascar bioproject PRJNA485056 SRA SRR7658642 from a 72-year-old adult fecal sample to SFB genomes of various hosts. Mapping of cleaned and trimmed reads was performed at 95% mapping stringency for nucleotide identity and read length for SFB genomes using the CLC genomic software package. QS: Quality score.

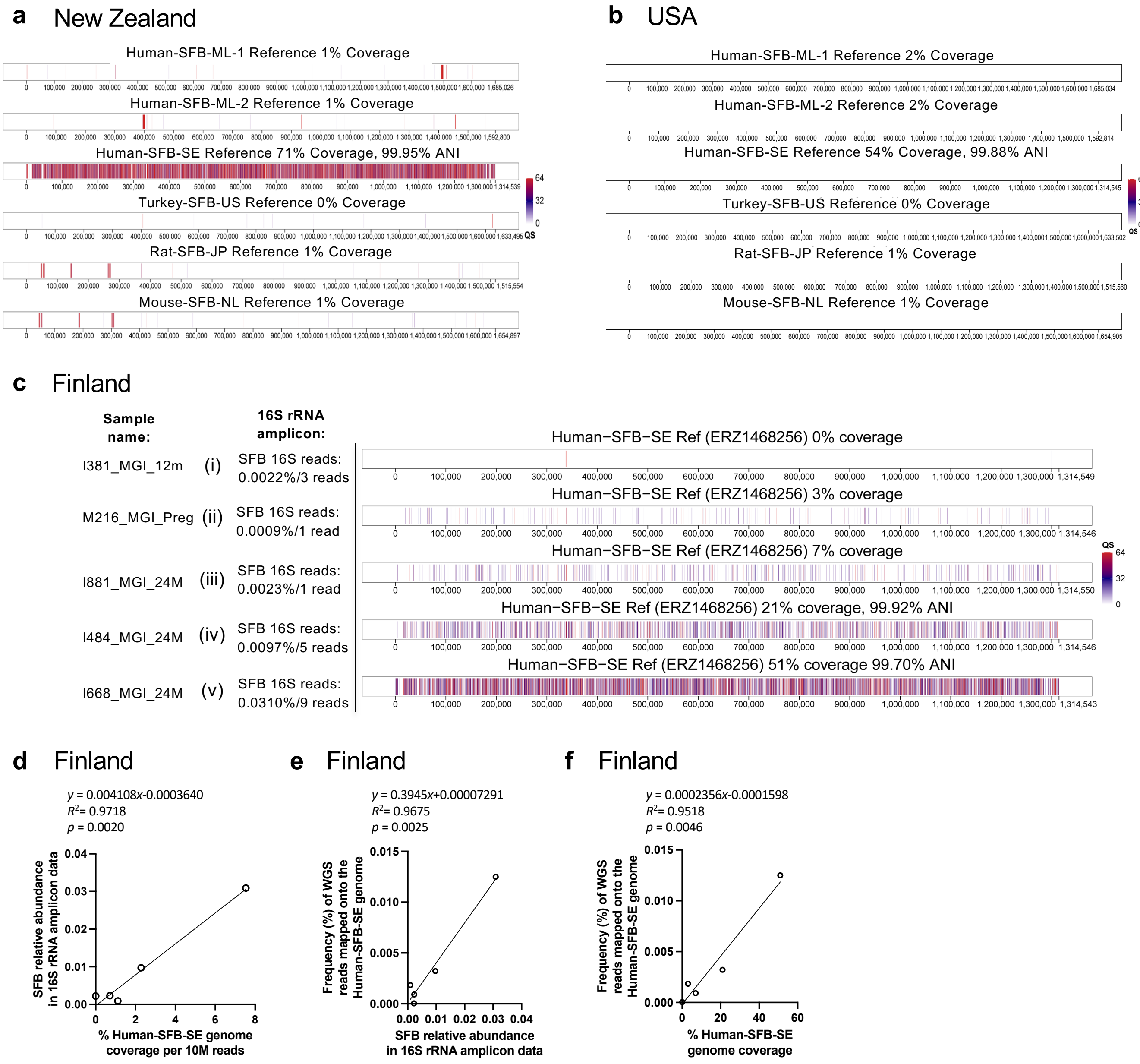

Supplementary Figure 26. SFB identification in metagenomic data from bioprojects originating in New Zealand, the USA, and Finland. a/b Mapping of trimmed sequence reads of **a** the bioproject PRJNA345144 SRA SRR7351900 from a 24-month-old New Zealand infant of European descent and **b** the bioproject PRJNA940472 SRA SRR23801710 from an American child to SFB genomes of various hosts. **c-f** Analysis of the Finnish bioproject PRJEB70237. **c** Mapping of trimmed metagenomic sequence reads from five (i-v) Finnish fecal samples, identified to be SFB-positive by 16S rRNA gene amplicon analysis of bioproject PRJEB55243, to the Human-SFB-SE genome; includes read frequency and number of the16S rRNA gene amplicon dataset. **d** Correlation between the percent Human-SFB-SE genome coverage in the Finnish bioproject PRJEB70237 per 10M reads compared to the SFB relative abundance in the equivalent samples from the 16S rRNA gene amplicon bioproject PRJEB55243. **e/f** Correlation between the frequency of reads from SRAs of bioproject PRJEB70237 mapped onto the Human-SFB-SE genome and the **e** relative abundance of SFB in the amplicon bioproject PRJEB55243 or **f** the coverage of the Human-SFB-SE genome sequence of the five SFB-positive samples. Read mapping on SFB genomes performed with a minimum 95% read length and 95% nucleotide identity using the CLC genomic software package. QS: Quality score.

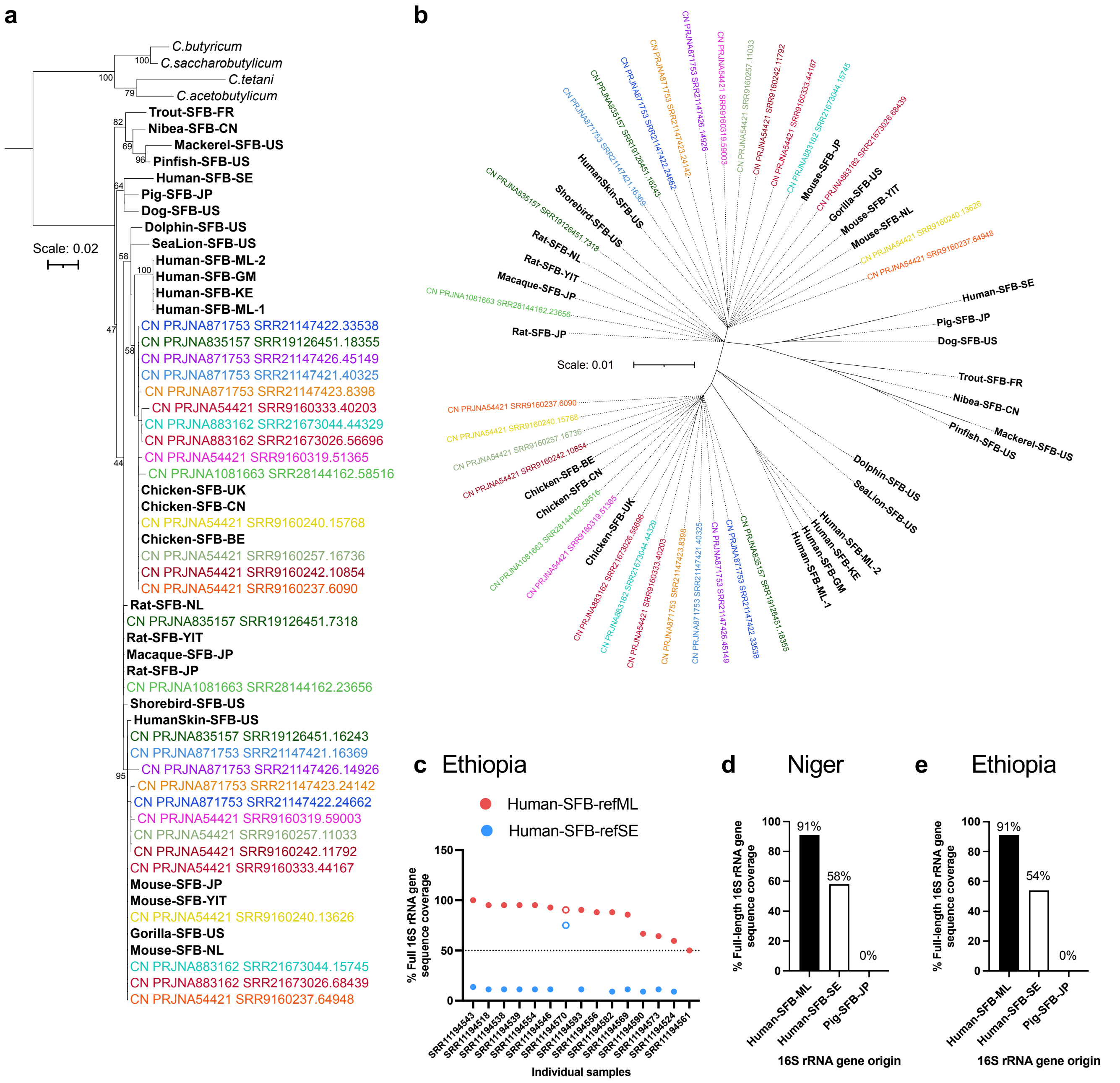

Supplementary Figure 27. Co-occurrence of two SFB lineages. a/b Maximum likelihood phylogenetic trees **a** with and **b** without *Clostridium* outgroups (italic) of SRAs from various bioprojects with two or more SFB V3-V4 16S rRNA gene sequences per SRA with the highest nucleotide identity to a separate SFB lineage. SFB 16S rRNA gene reads are labeled with their country of origin, using their two-letter code, the bioproject number, the SRA number, and the sequence read number. SFB 16S rRNA gene reads from the same sample are shown in the same color. Reference SFB 16S rRNA gene sequences are highlighted in bold. **c** SFB lineage co-occurrence within a sample for bioproject PRJNA608948 from Ethiopia based on RNA metagenomic sequencing data and percent 16S rRNA gene coverage using PebbleScout analysis. The open circle denotes the sample with Human-SFB-refSE and Human-SFB-refML co-occurrence. Samples on or above the dotted line are positive for the indicated human SFB lineage. **d/e** Percent 16S rRNA gene sequence coverage of Human-SFB-ML, Human-SFB-SE, and Pig-SFB-JP 16S rRNA gene sequences in samples with SFB lineage co-occurrence. **d** SRA SRR9843950 of bioproject PRJNA549968 from Niger; **e** SRA SRR11194570 of bioproject PRJNA608948 from Ethiopia. Percentages were obtained through mapping of paired-end RNA metagenomic reads using the CLC Genomic Workbench software. **a** Tree includes bootstrap values and for (**a/b**) the scale is nucleotide substitutions per nucleotide position.

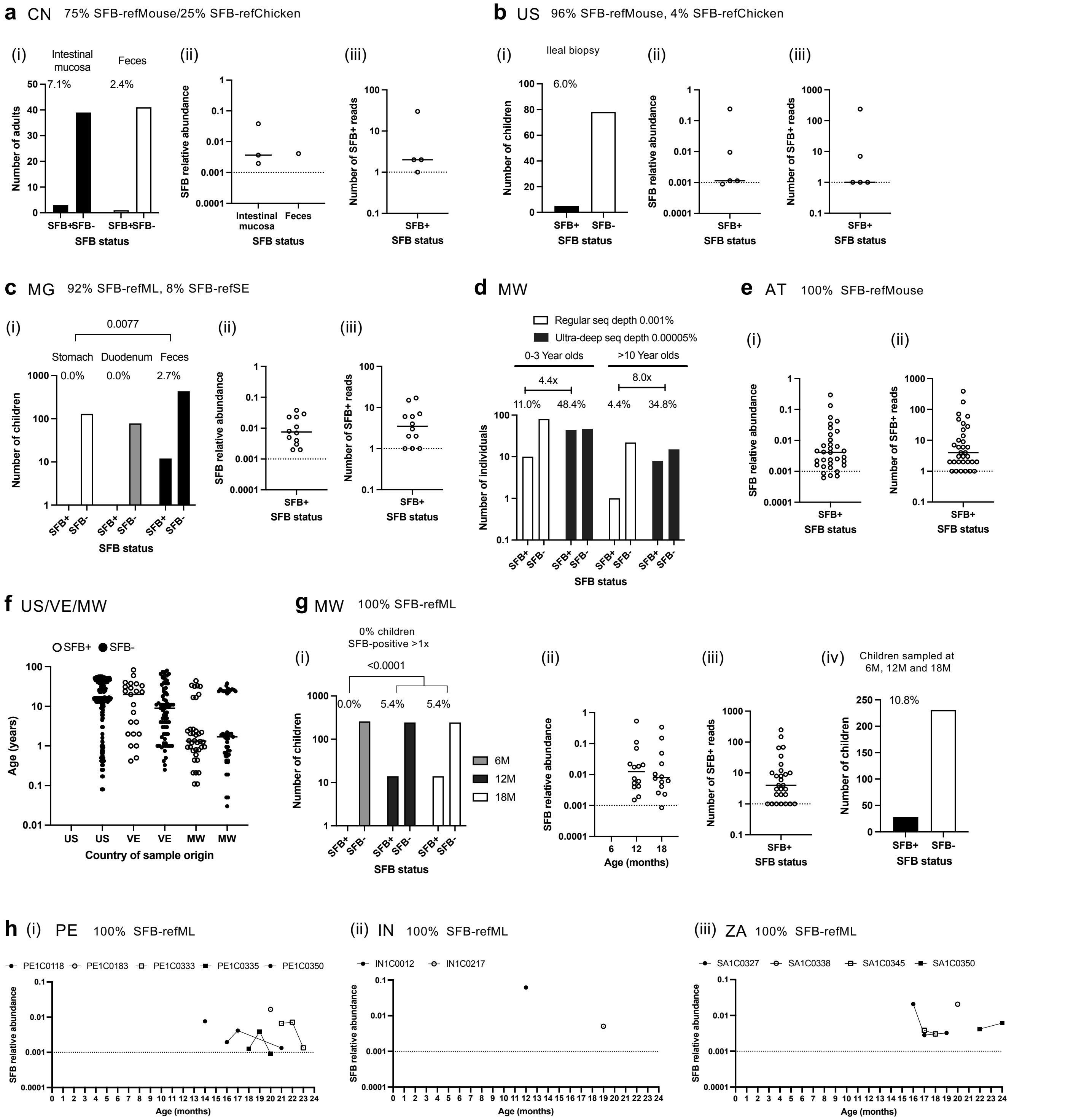
a CN 75% SFB-refMouse/25% SFB-refChicken
(i)
Intestinal mucosa
Feces
7.1%
2.4%
Number of adults
SFB+ SFB- SFB+ SFB-
SFB status
(ii)
SFB relative abundance
Intestinal mucosa
Feces
SFB status
(iii)
Number of SFB+ reads
SFB+
SFB status
b US 96% SFB-refMouse, 4% SFB-refChicken
(i)
Ileal biopsy
6.0%
Number of children
SFB+ SFB-
SFB status
(ii)
SFB relative abundance
SFB+
SFB status
(iii)
Number of SFB+ reads
SFB+
SFB status
c MG 92% SFB-refML, 8% SFB-refSE
(i)
0.0077
Stomach
Duodenum
Feces
0.0%
0.0%
2.7%
Number of children
SFB+ SFB- SFB+ SFB- SFB+ SFB-
SFB status
(ii)
SFB relative abundance
SFB+
SFB status
(iii)
Number of SFB+ reads
SFB+
SFB status
d MW
Regular seq depth 0.001%
Ultra-deep seq depth 0.00005%
0-3 Year olds
>10 Year olds
4.4x
8.0x
11.0%
48.4%
4.4%
34.8%
Number of individuals
SFB+ SFB- SFB+ SFB- SFB+ SFB- SFB+ SFB-
SFB status
e AT 100% SFB-refMouse
(i)
SFB relative abundance
SFB+
SFB status
(ii)
Number of SFB+ reads
SFB+
SFB status
f US/VE/MW
SFB+ SFB-
Age (years)
US US VE VE MW MW
Country of sample origin
g MW 100% SFB-refML
0% children SFB-positive >1x
(i)
<0.0001
0.0%
5.4%
5.4%
Number of children
6M
12M
18M
SFB+ SFB- SFB+ SFB- SFB+ SFB-
SFB status
(ii)
SFB relative abundance
6 12 18
Age (months)
(iii)
Number of SFB+ reads
SFB+
SFB status
(iv)
Children sampled at 6M, 12M and 18M
10.8%
Number of children
SFB+ SFB-
SFB status
h (i) PE 100% SFB-refML
PE1C0118
PE1C0183
PE1C0333
PE1C0335
PE1C0350
SFB relative abundance
Age (months)
(ii) IN 100% SFB-refML
IN1C0012
IN1C0217
SFB relative abundance
Age (months)
(iii) ZA 100% SFB-refML
SA1C0327
SA1C0338
SA1C0345
SA1C0350
SFB relative abundance
Age (months)

Supplementary Figure 28. Human SFB colonization location, relative abundance, and prevalence. a-c/f SFB 16S rRNA gene read analysis in **a,c-h** fecal samples and **b** ileal biopsies in various bioprojects (PRJEB/PRJNA). **a-c/f**(i) SFB prevalence (including the percent of SFB-positive individuals), **a-c/f**(ii) SFB relative abundance, and **a-c/f**(iii) SFB 16S rRNA gene read numbers. **a** PRJNA835157, a study of ulcerative colitis in China. Number of children: intestinal mucosa n=42, feces n=42. **b** PRJNA317429, a study of paediatric Crohn's disease in the USA. Number of children: biopsy n=83. **c** PRJEB48119, a study of malnutrition in children 2 to 5 years of age in Madagascar. Number of children: stomach n=130, duodenum n=78, feces n=448. **d** PRJEB3079, fecal samples from a study on microbiota diversity in Malawi. Prevalence of SFB highlighting the effect of ultra-deep sequencing versus regular sequencing depth on SFB prevalence calculation. **e** PRJEB55157, fecal samples in a study of elderberry supplementation in Austria with SFB **e**(i) relative abundance and **e**(ii) 16S rRNA read number. Number of individuals n=30, fecal samples n=264. **f** PRJEB3079, a study on microbiota diversity, including human fecal samples from Venezuela, Malawi, and the USA. Age of the individual at the time of sampling of SFB-positive and negative fecal samples. **g** PRJEB9853, gut microbiomes of Malawian twin pairs discordant for kwashiorkor; **g**(iv) SFB prevalence for all children sampled at 6, 12 and 18 months (M) time points. Number of children n=260 (at 6,12, and 18 n=259), number of samples n=776. **h** PRJEB27068, a longitudinal study of healthy and impaired human gut microbiota development in Bangladesh, Peru, India and South Africa. SFB relative abundance in fecal sample versus the age of the child at the time of sampling for **h**(i) Peru, **h**(ii) India and **h**(iii) South Africa. Number of children/fecal samples: Peru n=32/488, India n=14/331, and South Africa n=7/148**. a-h** Country of project origin is indicated by its 2-letter country code. **a-d,f-h** Includes is the percent of samples belonging to the indicated Human-SFB lineage. Crossbars indicate the median value of the datasets. A dotted line is included at **a-d/g**(ii) a relative abundance of 0.001% and **a-d/g**(iii) read number of 1 for reference purposes. **a-c,e/g** P-values were obtained with the two-sided Fisher's exact test.

Supplementary Table 1. General genome features of SFB genomes and draft Human-SFB-ML genome sequences generated by chromosome conformation capture (3C). a Analysis of the draft (3C) and final (ML) genome sequences of Human-SFB-ML-1 and 2. **b** Comparison of Human-SFB-ML genomes to genomes from human, avian and rodent origin.

a	HUMAN			
	Human-S340-3C	Human-S195-3C	Human-SFB-ML-1 (S340)	Human-SFB-ML-2 (S195)
Sequence size	1719939	1495531	1685034	1592805
Number of contigs	119	526	13	11
Shortest contig size	312	515	1232	6909
Median sequence size	1647	1896	67176	130348
Mean sequence size	14453,3	2843,2	129618	144800,5
Longest contig size	272868	21943	699134	392876
N50 value	63579	4435	233741	214765
N90	14203	1268	87382	94999
L50 value	7	103	2	3
L90	27	345	6	8
Size of contigs			699134, 233741, 215717, 178012, 121369, 87382, 67176, 41946, 16094, 13669, 6943, 2619, 1232	392876, 229049, 214765, 188268, 157790, 130348, 117682, 94999, 46655, 13464, 6909
# contigs (>= 0 bp)	119	526	13	11
# contigs (>= 1000 bp)	68	385	13	11
# contigs (>= 5000 bp)	33	82	11	11
# contigs (>= 10000 bp)	30	17	10	10
# contigs (>= 25000 bp)	21	0	8	9
# contigs (>= 50000 bp)	10	0	7	8
Total length (>= 0 bp)	1719939	1495531	1685034	1592805
Total length (>= 1000 bp)	1689196	1392257	1685034	1592805
Total length (>= 5000 bp)	1612772	652559	1681183	1592805
Total length (>= 10000 bp)	1593308	217165	1674240	1585896
Total length (>= 25000 bp)	1443934	0	1644477	1572432
Total length (>= 50000 bp)	1045155	0	1602531	1525777

b	HUMAN			AVIAN	RODENT			
	Human-SFB-ML-1	Human-SFB-ML-2	Human-SFB-SE	Turkey-SFB-US	Rat-SFB-YIT	Mouse-SFB-NL	Mouse-SFB-JP	Mouse-SFB-YIT
Genome Size (bp)	1685034	1592805	1314549	1633499	1515556	1654902	1620005	1586397
G+C content (%)	30,7	30,47	26,98	26,14	27,98	28,09	28,26	28,11
Genes	1696	1589	1318	1632	1462	1655	1540	1556
CDS	1592	1541	1276	1588	1397	1592	1607	1494
tRNAs	33	36	28	33	39	39	40	38
rRNAs	2	1	3	1	16	15	18	15
tmRNA	1	1	1	1	1	1	1	1
misc RNA	7	10	10	9	9	8	8	8
Hypothetical proteins	709	633	523	570	383	527	454	431
Proteins with F(x)al assignments	914	882	762	953	944	992	1010	988
Signal peptide	92	91	69	112	115	105	107	106
Isolate-specific proteins	48	19	201	46	56	60	25	3
% Completeness CheckM v1.1.0	99,85	99,74	85,91	99,48	99,85	99,27	99,29	99,76
% Contamination CheckM v1.1.0	0,62	1,02	0	0,3	0,11	0,39	0,42	0,31
Contigs	13	11	153	41	1	1	1	1

Supplementary Table 2. Whole-genome average nucleotide and amino acid identity analysis of SFB from phylogenetically distant host species. a Average nucleotide identity (ANI) was calculated using FastANI. **b** Average amino acid identity (AAI) was calculated using EzAAI. Values are color scaled from highest (red) to middle (orange) to lowest (green) values.

a

Whole-genome Average Nucleotide Identity (ANI)								
	Human-SFB-ML-1	Human-SFB-ML-2	Human-SFB-SE	Turkey-SFB-US	Rat-SFB-YIT	Mouse-SFB-NL	Mouse-SFB-JP	Mouse-SFB-YIT
Human-SFB-ML-1	100,0	99,5	77,6	78,5	77,3	77,8	77,7	77,3
Human-SFB-ML-2	99,5	100,0	77,5	78,3	77,2	77,8	77,5	77,3
Human-SFB-SE	77,1	77,7	100,0	78,7	77,4	77,6	77,7	77,5
Turkey-SFB-US	78,4	78,2	78,6	100,0	78,4	78,6	78,5	78,5
Rat-SFB-YIT	77,2	77,3	77,4	78,3	100,0	85,2	85,2	85,2
Mouse-SFB-NL	77,9	77,6	77,6	78,6	85,3	100,0	99,6	99,6
Mouse-SFB-JP	77,6	77,4	77,6	78,6	85,2	99,6	100,0	99,9
Mouse-SFB-YIT	77,5	77,4	77,4	78,6	85,2	99,6	99,9	100,0

b

Whole-genome Average Amino Acid Identity (AAI)								
	Human-SFB-ML-1	Human-SFB-ML-2	Human-SFB-SE	Turkey-SFB-US	Rat-SFB-YIT	Mouse-SFB-NL	Mouse-SFB-JP	Mouse-SFB-YIT
Human-SFB-ML-1	100,0	99,3	67,4	72,9	66,6	67,1	67,0	66,9
Human-SFB-ML-2	99,2	100,0	67,4	73,0	66,7	67,0	67,1	67,0
Human-SFB-SE	67,4	67,4	100,0	71,9	67,2	67,1	67,1	67,2
Turkey-SFB-US	73,0	72,9	71,9	100,0	70,4	70,8	71,2	71,0
Rat-SFB-YIT	66,7	66,7	67,2	70,7	100,0	83,8	83,9	83,8
Mouse-SFB-NL	67,2	67,0	67,1	71,2	83,9	100,0	99,4	99,4
Mouse-SFB-JP	67,0	67,1	67,1	71,1	83,9	99,4	100,0	99,7
Mouse-SFB-YIT	66,8	67,0	67,2	71,0	83,6	99,4	99,7	100,0

Supplementary Table 3. Human-SFB-ML has a unique set of transporters. Overview of the presence (x/grey) or absence of genes within the PTS or ABC transporter category in Human-SFB-ML and in SFB from various host species. Analysis performed using the Kyoto Encyclopedia of Genes and Genomes (KEGG) database; ABC transporter list is non-exhaustive. Presence of a predicted factor is indicated by an x in a shaded box. Factors in the same row are in the same gene cluster.

PTS TRANSPORTERS									
	Homolog	Human-SFB-ML-1	Human-SFB-ML-2	Human-SFB-SE	Turkey-SFB-US	Rat-SFB-YIT	Mouse-SFB-NL	Mouse-SFB-JP	Mouse-SFB-YIT
Phosphoenol-pyruvate Family									
Phosphoenol-pyruvate	PtsI	x	x	x	x	x	x	x	x
	PtsH	x	x	x	x	x	x	x	x
Glucose Family									
Glucose	PtsG			x	x				
	Crr			x	x				
Maltose/Glucose	MalX			x	x				
	Crr			x	x				
Sucrose	ScrA				x				
Ph-β-Glucoside	BglF			x			x	x	x
Fructose Family									
Mannitol	MtlA	x	x	x	x	x	x	x	x
Fructose	FruA/B				x	x	x	x	x
	FuK				x	x	x	x	x
Lactose Family									
Cellobiose/Diacetylchitobiose	CelB	x	x	x	x	x	x	x	x
	CelC	x	x	x	x	x	x	x	x
	CelA	x	x	x	x	x	x	x	x
Mannose Family									
Mannose	ManY	x	x		x	x	x	x	x
	ManZ	x	x		x	x	x	x	x
	ManX	x	x		x	x	x	x	x
L-Ascorbate Family									
L-Ascorbate	UlaA			x	x	x	x	x	x
	UlaC			x	x	x	x	x	x
	UlaB			x	x	x	x	x	x

ABC TRANSPORTERS									
	Homolog	Human-SFB-ML-1	Human-SFB-ML-2	Human-SFB-SE	Turkey-SFB-US	Rat-SFB-YIT	Mouse-SFB-NL	Mouse-SFB-JP	Mouse-SFB-YIT
Mineral and Organic Ion Transporters									
Alkanesulfonate	SsuA	x	x						
	SsuC	x	x						
	SsuB	x	x						
Osmoprotectant	OpuBC	x	x						
	OpuBB	x	x						
	OpuBA	x	x						
Oligosaccaride, Polyol and Lipid Transporters									
Maltooligosaccharide	MdxE	x	x						
	MdsF	x	x						
	MdxG	x	x						
	MsmX	x	x		x	x	x	x	x
Aldouronate	LplA				x	x	x	x	x
	LplB				x	x	x	x	x
	LplC				x	x	x	x	x
Nucleoside	BmpA	x	x	x	x	x			
	NupB			x	x	x			
	NupC			x	x	x			
	NupA			x	x	x			
Monosaccharide Transporters									
Ribose/Autoinducer-2/D-Xylose	RbsB			x		x	x	x	x
	RbsC			x		x	x	x	x
	RbsD			x		x	x	x	x
	RbsA			x		x	x	x	x
Phosphate and Amino Acid Transporters									
Phosphate	PstS	x	x	x	x	x	x	x	x
	PstC	x	x		x	x	x	x	x
	PstA	x	x	x	x	x	x	x	x
	PstB	x	x	x	x	x	x	x	x
Phosphonate	PhnD				x	x	x	x	x
	PhnE				x	x	x	x	x
	PhnC				x	x	x	x	x
Arginine/Lysine/Histidine	ArtP	x	x	x	x	x	x	x	x
	ArtQ	x	x	x	x	x	x	x	x
	ArtR	x	x	x	x	x	x	x	x
Metallic Cation and Vitamin Transporters									
Zinc	ZnuA	x	x	x	x	x	x	x	x
	ZnuB	x	x	x	x	x	x	x	x
	ZnuC	x	x	x	x	x	x	x	x
Ferrous iron	FeoA	x	x	x	x	x	x	x	x
	FeoB	x	x	x	x	x	x	x	x
Ferrichrome	FhuD	x	x	x	x	x	x	x	x
	FhuB	x	x	x	x	x	x	x	x
	FhuG	x	x	x	x	x	x	x	x
	FhuA	x	x	x	x	x	x	x	x
Ferric iron	YclQ	x	x	x	x	x	x	x	x
	YclP	x	x	x	x	x	x	x	x
	YclO	x	x	x	x	x	x	x	x
	YclN	x	x	x	x	x	x	x	x
Biotin	BioY					x	x	x	x
	EcfT	x	x	x	x	x	x	x	x
	EcfA1	x	x	x	x	x	x	x	x
	EcfA2	x	x	x	x	x	x	x	x
Cobalamin	BtuF	x	x	x	x	x	x	x	x
	BtuC	x	x	x	x	x	x	x	x
	BtuD	x	x	x	x	x	x	x	x
Other ABC Transporters									
LANTIBIOTICS	NisE	x							
	NisG	x							
	NisF	x							

Supplementary Table 4. Characterization of the binding interaction of MdxE from Human-SFB-ML with a range of sugars. MdxE-sugar binding affinity (KD) to simple and oligomeric sugars was determined using a Biacore® system and a range of sugar concentrations in duplicate experiments. N/A: Not Applicable.

	KD (mM)	Sugar concentration range (mM)
Glucose	6,05E+00	8,23E-01 - 2,22E+01
Trehalose	N/A	5,00E+00
Isomaltose	N/A	5,00E+00
Maltose	2,06E-02	3,29E-03 - 8,00E-01
Maltotriose	7,17E-05	6,17E-06 - 1,50E-03
Maltotetraose	1,31E-04	6,17E-06 - 1,50E-03
Maltopentaose	1,54E-04	6,17E-06 - 1,50E-03
Maltohexaose	1,00E-04	6,17E-06 - 1,50E-03
Maltoheptaose	1,24E-04	6,17E-06 - 1,50E-03
α-Cyclodextrin	5,86E-02	1,03E-03 - 2,50E-01
γ-Cyclodextrin	7,42E-03	1,03E-04 - 2,50E-02
Sucrose	N/A	5,00E+00

Supplementary Table 5. Human-SFB-ML has a unique set of genes potentially related to reducing the impact of environmental stresses. Overview of the presence (x/grey) or absence of genes or gene families present in human SFB and in SFB from various other host species that are potentially linked to resisting environmental stresses. Presence of a predicted factor is indicated by an x in a shaded box. Factors in the same row are in the same gene cluster.

**Oxygen-related Stress**								
	Human-SFB-ML-1	Human-SFB-ML-2	Human-SFB-SE	Turkey-SFB-US	Rat-SFB-YIT	Mouse-SFB-NL	Mouse-SFB-JP	Mouse-SFB-YIT
Peroxiredoxin (AhpC)				x				
Peroxiredoxin	x	x		x	x	x	x	x
Thioredoxin	x	x	x	x	x	x	x	x
Thioredoxin				x				
Thioredoxin	x	x						
Thioredoxin reductase	x	x	x	x	x	x	x	x
Thioredoxin reductase						x	x	x
Catalase (manganese-containing)	x	x			x	x	x	x
Catalase				x	x	x	x	x
Rubredoxin/Flavodoxin	x	x	x	x	x	x	x	x
Rubredoxin/Rubrerythrin	x	x	x	x	x	x	x	x
Rubrerythrin	x	x		x	x	x	x	x
Rubrerythrin			x	x	x	x	x	x
Rubrerythrin						x	x	x
Glutathione peroxidase	x	x						
Glutaredoxin-related protein	x	x	x	x		x	x	x
Peroxide stress protein (YaaA)	x	x		x	x	x	x	x
Peroxide responsive repressor	x	x						
Hydroperoxide resistance tc regulator (ohrR)	x	x						
Flavodoxin	x	x						
Flavodoxin	x							
**Nitrogen-related Stress**								
	Human-SFB-ML-1	Human-SFB-ML-2	Human-SFB-SE	Turkey-SFB-US	Rat-SFB-YIT	Mouse-SFB-NL	Mouse-SFB-JP	Mouse-SFB-YIT
Arginase	x	x	x	x	x	x	x	x
Agmatinase	x	x						
Nitroreductase	x	x						
Nitroreductase	x	x						
Nitroreductase	x	x						
Nitroreductase (O2-insensitive NADPH)				x				
**Oxidoreductases**								
	Human-SFB-ML-1	Human-SFB-ML-2	Human-SFB-SE	Turkey-SFB-US	Rat-SFB-YIT	Mouse-SFB-NL	Mouse-SFB-JP	Mouse-SFB-YIT
NADPH-dependent FMN reductase	x	x						
NADPH-dependent FMN reductase				x				
NAD(P)H dehydrogenase (quinone)	x	x						
NAD(P)H dehydrogenase (quinone)				x				
NADH:flavin oxidoreductase	x	x						
NADH:flavin oxidoreductase					x	x	x<br>x	x<br>x
Short-chain dehydrogenase/reductase	x	x						
Putative short-chain dehydrogenase/reductase	x	x						
Short-chain dehydrogenase/reductase			x					

Supplementary Table 6. Analysis of the *Peptoclostridium difficile* genome BN1097 contig 93. Identification of the contig93 in the BN1097 draft genomic sequence with uncharacteristically high (100%) nucleotide identity to the Mouse-SFB-NL (CP008713) genome used as the alignment reference genome. Contig93 is composed of the 16, 23, and 5 rRNA genes.

Query	Subject	Contig	Query Start	Query Stop	Query Strand	Subject Start	Subject Stop	Subject Strand	Length	Identity	Coverage	Mismatches	Gaps	Gap Bases	Score	E-Value
CP008713,1	LK932435,1	93	184089	188426	+	9	4347	+	4338	99,9769	0,26213	1	0	0	0	0
CP008713,1	LK932435,1	93	299889	304321	+	1	4347	+	4346	99,977	0,262614	1	0	0	0	0
CP008713,1	LK932435,1	93	304906	309244	+	9	4347	+	4338	99,977	0,262191	1	0	0	0	0
CP008713,1	LK932435,1	93	51395	55732	+	9	4347	+	4338	100	0,26213	0	0	0	0	0
CP008713,1	LK932435,1	93	43168	47505	+	9	4347	+	4338	100	0,26213	0	0	0	0	0

Supplementary Table 7. Co-colonization of SFB lineages based on the percent nucleotide identity of reads covering the V3-V4 16S rRNA gene sequence region. Identification of multiple SFB lineages in a single fecal sample. Phylogenetic positioning of the SFB lineage reference reads for each sample with multiple SFB lineages is shown in Supplementary Fig. 27.

Country	Bioproject	SRA	Full SRA name	Reference SFB 16S rRNA gene			Total Reads	% Chicken-SFB-BE	% Mouse-SFB-NL	% Rat-SFB-JP	Median read length (bp) Chicken-SFB-BE	Median read length (bp) Mouse-SFB-NL	Median read length (bp) Rat-SFB-JP
				Chicken-SFB-BE	Mouse-SFB-NL	Rat-SFB-JP							
China	PRJNA1081663	SRR28144162	CN_PRJNA1081663_SRR28144162	1	0	2	3	33	0	67	445		445
	PRJNA544721	SRR9160237	CN_PRJNA544721_SRR9160237	7	9	0	16	44	56	0	426	426	
	PRJNA544721	SRR9160240	CN_PRJNA544721_SRR9160240	9	3	0	12	75	25	0	426	426	
	PRJNA544721	SRR9160242	CN_PRJNA544721_SRR9160242	7	2	0	9	78	22	0	426	426	
	PRJNA544721	SRR9160257	CN_PRJNA544721_SRR9160257	3	1	0	4	75	25	0	426	426	
	PRJNA544721	SRR9160319	CN_PRJNA544721_SRR9160319	4	2	0	6	67	33	0	426	426	
	PRJNA544721	SRR9160333	CN_PRJNA544721_SRR9160333	1	12	0	13	8	92	0	426	426	
	PRJNA835157	SRR19126451	CN_PRJNA835157_SRR19126451	1	28	1	30	3	93	3	405	405	405
	PRJNA871753	SRR21147421	CN_PRJNA871753_SRR21147421	1	4	0	5	20	80	0	402	402	
	PRJNA871753	SRR21147422	CN_PRJNA871753_SRR21147422	2	1	0	3	67	33	0	402	402	
	PRJNA871753	SRR21147423	CN_PRJNA871753_SRR21147423	1	1	0	2	50	50	0	402	402	
	PRJNA871753	SRR21147426	CN_PRJNA871753_SRR21147426	1	1	0	2	50	50	0	402	402	
	PRJNA883162	SRR21673026	CN_PRJNA883162_SRR21673026	3	4	0	7	43	57	0	445	445	
	PRJNA883162	SRR21673044	CN_PRJNA883162_SRR21673044	1	2	0	3	33	67	0	445	445	
