# Supplementary material for: Segmented filamentous bacteria are worldwide human gut commensals
Source: Nat Commun. 2026 Mar 5;17:4174. doi: 10.1038/s41467-026-70010-4 (PMC13153320; doi:10.1038/s41467-026-70010-4)
Supplement: Supplementary file 2 — Description of Additional Supplementary Files [file 41467_2026_70010_MOESM2_ESM.docx]

**Kiran S *et al.* 2026 Nature Communications**

**Segmented filamentous bacteria are worldwide human gut commensals**

**Supplementary Data 1. SFB 16S rRNA gene sequence nucleotide identity across SFB from various hosts and compared to related *Clostridium* species.** The nucleotide sequence identity for the **a** 1470 bp trimmed full-length, **b** 1366 bp trimmed full-length, **c** V1-V2, **d** V3-V4, **e** V4, and **f** V1-V4 regions of the 16S rRNA gene of SFB from various hosts. For **a/b** actual sequence lengths may be shorter (Supplementary Fig. 3). 16S rRNA gene sources: Human-SFB-ML-1_PX000509 and ML-2_PX000510 (this study), Human-SFB-KE_PX000511 (this study), Human-SFB-GM_PX000512 (this study), Human-SFB-SE contig141_89555, HumanSkin-SFB-US_JF168221.1, Pig-SFB-JP_AB822980.1, Chicken-SFB-BE_PV993571.1, Chicken-SFB-UK_X80834.1, Chicken-SFB-CN_DQ342328.1, Chicken-SFB-CN_DQ342328.1, Shorebird-SFB-US_KC478326.1, Mouse-SFB-NL_CP008713.1, Mouse-SFB-JP_AP012202.1, Mouse-SFB-YIT_AP012209.1, Rat-SFB-NL_X87244.1, Rat-SFB-JP_D86302.1, Rat-SFB-YIT_AP012210.1, Macaque-SFB-JP_D86303.1, Gorilla-SFB-US_EU474247.1, Dog-SFB-US_DQ113757.1, Sealion-SFB-US_JQ207968.1, Dolphin-SFB-US_JQ202596.1, Mackerel-SFB-US_JQ191772.1, Pinfish-SFB-US_KJ197471.1, Trout-SFB-FR_AY007720.1 and Nibea-SFB-CN_KX431301.1. Sequences are identified by their country of origin, indicated by the country 2-letter code, and the NCBI Genbank number, when available. Four *Clostridium* species closely related to SFB are included for comparison: *C. amylolyticum* (NR_074511), *C. saccharobutylicum* (NR_122051), *C. butyricum* (CP040626), and *C. tetani* (X74770). Values are color scaled from highest (red) to middle (orange) to lowest (green) values.

**Supplementary Data 2. BSR score ratio analysis of Human-SFB-ML genomes compared to complete, draft, and read-mapped SFB genome consensus genomes from various hosts and locations.** Reference genomes are the concatemerized genome of **a** Human-SFB-ML-1 and **b** Human SFB-ML-2. Values are color scaled from highest (red) to middle (white) to lowest (green) values.

**Supplementary Data 3. Gene Category (GC) and Cluster of Orthologous Gene (COG) annotation of SFB genomes from various hosts.** SFB genome sequences include Human-SFB-ML-1 (JBRACM000000000, this study), Human-SFB-ML-2 (JBRACM000000001, this study) and the genomes of Rat-SFB-YIT (AP012210), Mouse-SFB-NL (CP008713), Mouse-SFB-JP (AP012202), Mouse-SFB-YIT (AP012209), Turkey-SFB-US (UMNCA01), and Human-SFB-SE (ERZ1468256).

**Supplementary Data 4. Summary of bioinformatic identification of SFB 16S rRNA gene amplicon reads in publicly available datasets.** Projects with SFB 16S rRNA sequence-positive hits are grouped by country of origin of sample and referenced based on the NCBI nomenclature. A bioproject is listed multiple times when samples originated from different countries. The original publication is provided when available (DOI). The dominant reference SFB per bioproject is provided based on either SRA or total 16S rRNA gene read-based analysis, as well as based on the dominant sequence. The reference SFB assigned to the dominant sequence of each project is indicated. Values are color scaled from highest (red) to middle (orange) to lowest (green) values. SFB 16S rRNA gene reference sequences are color coded for clarity.

**Supplementary Data 5. Summary of bioinformatic identification of SFB in 16S rRNA gene multivariable amplicon datasets and RNA and genomic-based metagenomic sequencing datasets.** Projects with SFB-positive reads are grouped by country of origin of sample and referenced based on the NCBI nomenclature.

**Supplementary Data 6. WGS and multiplex samples positive for SFB 16S rRNA gene reads.** Samples are color-coded based on their SFB lineage designation (Human-SFB-refML: red; Human-SFB-refSE: blue) or the co-occurrence of Human-SFB-refML and Human-SFB-refSE (purple).

**Supplementary Data 7. Summary of SRA-based bioinformatic analysis per country.** Projects with SFB-positive samples covering the V4, V3-V4, or V1-V2 region. SRAs originating from the same country were grouped and classified based on one of the four SFB 16S rRNA gene references to which the majority of its SFB reads had the highest sequence identity. The percentage of SRA per country for each reference SFB is indicated as well as the dominant human SFB lineage per country. Reads from SRAs belonging to the dominant human SFB lineage were analyzed in terms of their maximum read identity to the SFB reference strain as well as their read length. The single sequence for Singapore and Saudi Arabia were identified in NCBI Genbank database. All analyzed 16S rRNA gene amplicon projects, except for Venezuela, include 16S rRNA gene reads greater or equal to 200 bp. Included are also countries with bioprojects based on 16S rRNA gene multiplex PCR or RNA metagenomics. Bioprojects based on DNA metagenomic analysis are only included for countries without RNA-based analysis, except France where the metagenomic analysis identified an additional SFB lineage not found in the 16S rRNA gene amplicon datasets. Values are color scaled from highest (red) to middle (orange) to lowest (green) values. SFB 16S rRNA gene reference sequences are color coded for clarity.

**Supplementary Data 8. Percent nucleotide identity of the reference 16S rRNA gene read per bioproject to the SFB 16S rRNA gene sequence of various hosts.** Nucleotide identity between the dominant SFB 16S rRNA gene read of each bioproject and the four SFB 16S rRNA reference gene sequences from varying hosts for the 16S variable regions V1-V2, V4, and V3-V4, when available. Values are color scaled from highest (red) to middle (orange) to lowest (green) values.
