# Supplementary material for: Segmented filamentous bacteria are worldwide human gut commensals
Source: Nat Commun. 2026 Mar 5;17:4174. doi: 10.1038/s41467-026-70010-4 (PMC13153320; doi:10.1038/s41467-026-70010-4)
Supplement: Supplementary file 4 — Reporting Summary [file 41467_2026_70010_MOESM4_ESM.pdf]

nature portfolio

Corresponding author(s): Pamela Schnupf

Last updated by author(s): 22/1/2026

Reporting Summary

Nature Portfolio wishes to improve the reproducibility of the work that we publish. This form provides structure for consistency and transparency in reporting. For further information on Nature Portfolio policies, see our Editorial Policies and the Editorial Policy Checklist.

Statistics

For all statistical analyses, confirm that the following items are present in the figure legend, table legend, main text, or Methods section.

n/a	Confirmed	
☐	☒	The exact sample size (*n*) for each experimental group/condition, given as a discrete number and unit of measurement
☐	☒	A statement on whether measurements were taken from distinct samples or whether the same sample was measured repeatedly
☐	☒	The statistical test(s) used AND whether they are one- or two-sided<br>*Only common tests should be described solely by name; describe more complex techniques in the Methods section.*
☒	☐	A description of all covariates tested
☒	☐	A description of any assumptions or corrections, such as tests of normality and adjustment for multiple comparisons
☐	☒	A full description of the statistical parameters including central tendency (e.g. means) or other basic estimates (e.g. regression coefficient) AND variation (e.g. standard deviation) or associated estimates of uncertainty (e.g. confidence intervals)
☐	☒	For null hypothesis testing, the test statistic (e.g. *F*, *t*, *r*) with confidence intervals, effect sizes, degrees of freedom and *P* value noted<br>*Give P values as exact values whenever suitable.*
☒	☐	For Bayesian analysis, information on the choice of priors and Markov chain Monte Carlo settings
☒	☐	For hierarchical and complex designs, identification of the appropriate level for tests and full reporting of outcomes
☒	☐	Estimates of effect sizes (e.g. Cohen's *d*, Pearson's *r*), indicating how they were calculated

Our web collection on statistics for biologists contains articles on many of the points above.

Software and code

Policy information about availability of computer code

Data collection	Raw sequence data were generated using standard sequencing platforms, and additional publicly available data were download from NCBI and ENA. No custom software was used for data collection.
Data analysis	All analyses were conducted using standard bioinformatics and statistical tools described in the Materials and Methods section. No new software or custom algorithms were developed.<br>List of tools used (Methods): Data Preprocessing and Quality Control<br>-Trimmomatic v0.36: Quality control and trimming of Illumina reads<br>-Cutadapt v2.10: Adapter and quality trimming<br>-FastQC v0.11.9: Quality assessment of sequencing data<br>-FASTP v0.23.4: FASTQ quality control and preprocessing<br>-FLASH2 v2.2.11: Merging paired-end reads<br>Read Filtering and Mapping<br>-BMTagger v3.101: Removal of human-origin reads<br>-Bowtie v1: rRNA removal by mapping to SILVA database<br>-CLC Genomics Workbench v23.0.5: Read mapping and consensus sequence generation<br>-NCBI BLAST v2.14.1+: Local nucleotide-nucleotide alignment (blastn) of amplicon sequencing reads against custom 16S rRNA gene database<br>Assembly and Scaffolding<br>-metaSPAdes v3.11: Illumina metagenome assembly<br>-Canu v1.8: PacBio long-read assembly<br>-MetaTOR pipeline v1.1.0: Meta3C binning and assembly

-GRAAL algorithm: Genome scaffolding
-SSPACE v2.1.1: Contig scaffolding
-GapFiller: Filling gaps in scaffolds
Annotation and Functional Analysis
-Prokka v1.14.6: Prokaryotic genome annotation
-Anvi'o v6.2: Pangenome analysis and gene clustering
-eggNOG-mapper v2 / eggNOG database v5.0: Protein functional annotation
-Pfam v34.0: Protein family annotation
-KEGG mapper/BRITE, GhostKOALA: Metabolic pathway annotation
-mobileOG-db v1.1.3: Mobile genetic element annotation
Phylogenetic and Genomic Analysis
-EzAAI v1.2.1: Average amino acid identity analysis
-FastANI v1.34: Average nucleotide identity analysis
-IQ-TREE2: Maximum-likelihood phylogenetic analysis
-trimAl: Phylogenetic alignment trimming
-MAFFT v7.0: Multiple sequence alignment
-SimiPlot v1.1: Genomic similarity plotting
-PebbleScout,NCBI: identification of public datasets containing SFB 16S rRNA gene
Statistical and Visualization Tools
-GraphPad Prism 9.1.0: Statistical analysis
-R v4.0.2 (with ggplot2, patchwork, Biostrings, SeqinR, UpSetR, rnaturalearth, countrycode, ggrepel, tidyverse packages): Data analysis and visualization
-iTOL: Phylogenetic tree visualization
-Proksee: Genome characterization and visualization
Experimental Data Analysis Software
-Biacore T200 Evaluation Software v3.1: Surface plasmon resonance (SPR) binding affinity analysis
Statistical analysis:
Contingency: Two-sided Fisher's exact test with Prism v10.6.1
Conditions: Two-sided Mann Whitney exact test Prism v10.6.1

For manuscripts utilizing custom algorithms or software that are central to the research but not yet described in published literature, software must be made available to editors and reviewers. We strongly encourage code deposition in a community repository (e.g. GitHub). See the Nature Portfolio guidelines for submitting code & software for further information.

Data

Policy information about availability of data

All manuscripts must include a data availability statement. This statement should provide the following information, where applicable:

- Accession codes, unique identifiers, or web links for publicly available datasets
- A description of any restrictions on data availability
- For clinical datasets or third party data, please ensure that the statement adheres to our policy

DATA AVAILABILITY
The raw sequencing files and the metagenome-assembled genomes (MAGs) for Human-SFB-ML-1 and ML-2 generated in this study have been deposited in the National Center for Biotechnology Information (NCBI) database under bioproject PRJNA1106451 and includes the genome assemblies with accession numbers JBRACM000000000 (Human-SFB-ML-1) and JBRACM000000001 (Human-SFB-ML-2). The 16S rRNA gene sequences are made available at NCBI under the GenBank accession numbers PX000509 (Human-SFB-ML-1), PX000510 (Human-SFB-ML-2), PX000511 (Human-SFB-KE), and PX000512 (Human-SFB-GM).
Additional SFB 16S rRNA gene sequences used in this study: Human-SFB-SE [contig141_89555], HumanSkin-SFB-US [JF168221.1], Pig-SFB-JP [AB822980.1], Chicken-SFB-BE [PV993571.1], Chicken-SFB-UK [X80834.1], Chicken-SFB-CN [DQ342328.1], Chicken-SFB-CN [DQ342328.1], Shorebird-SFB-US [KC478326.1], Mouse-SFB-NL [CP008713.1], Mouse-SFB-JP [AP012202.1], Mouse-SFB-YIT [AP012209.1], Rat-SFB-NL [X87244.1], Rat-SFB-JP [D86302.1], Rat-SFB-YIT [AP012210.1], Macaque-SFB-JP [D86303.1], Gorilla-SFB-US [EU474247.1], Dog-SFB-US [DQ113757.1], Sealion-SFB-US [JQ207968.1], Dolphin-SFB-US [JQ202596.1], Mackerel-SFB-US [JQ191772.1], Pinfish-SFB-US [KJ197471.1], Trout-SFB-FR [AY007720.1] and Nibea-SFB-CN [KX431301.1]. Four Clostridium species used as outgroups for phylogenetic analysis: C. acetobutylicum [NR_074511], C. saccharobutylicum [NR_122051], C. butyricum [CP040626], and C. tetani [X74770.1]. SFB genome sequences used for the comparative analysis are: Rat-SFB-YIT [AP012210], Mouse-SFB-NL [CP008713], Mouse-SFB-JP [AP012202], Mouse-SFB-YIT [AP012209], Turkey-SFB-US [UMNCA01], and Human-SFB-SE [ERZ1468256]. The Turkey-SFB-US 16S rRNA gene sequence in Supplementary Figures 3 and 7 was obtained from the assembled genome sequence. Source data are provided for the analysis of new data generated in this study and presented in Figures 1 to 6 and Supplementary Figures 13, 15, 23, 25 and 26. All bioprojects analyzed in this study are listed in Supplementary data 4 and 5.

The datasets included in this study are: PRJNA1048169, PRJDB10526, PRJDB10530, PRJDB12212, PRJEB20354, PRJEB21504, PRJEB23120, PRJEB24771, PRJEB24843, PRJEB27068, PRJEB27868, PRJEB28159, PRJEB29421, PRJEB29433, PRJEB3079, PRJEB3079, PRJEB31684, PRJEB3324, PRJEB34168 , PRJEB38986, PRJEB40425, PRJEB40986, PRJEB43871, PRJEB46240, PRJEB48119, PRJEB49206, PRJEB51728, PRJEB55157, PRJEB55243, PRJEB70237, PRJEB9818, PRJEB9853, PRJNA1029396, PRJNA1031121, PRJNA1045584, PRJNA1059486, PRJNA1081663, PRJNA1137832, PRJNA234437, PRJNA237362, PRJNA284397, PRJNA300541, PRJNA317429, PRJNA328966, PRJNA345144 , PRJNA392458, PRJNA393083, PRJNA436359, PRJNA445763, PRJNA453621, PRJNA483304, PRJNA483523, PRJNA485056, PRJNA486712, PRJNA487683, PRJNA506262, PRJNA517050, PRJNA539933, PRJNA541075, PRJNA544721, PRJNA547591, PRJNA549968, PRJNA561510, PRJNA574920, PRJNA589343, PRJNA608948, PRJNA629760, PRJNA631204, PRJNA642859, PRJNA642975, PRJNA670523, PRJNA678145, PRJNA690835, PRJNA690972, PRJNA701749, PRJNA727842, PRJNA739008, PRJNA748675, PRJNA750255, PRJNA769052, PRJNA769228, PRJNA784725, PRJNA786061, PRJNA800628, PRJNA814846, PRJNA818501, PRJNA824552, PRJNA835157, PRJNA871753, PRJNA873936, PRJNA883162, PRJNA938107, PRJNA940472, PRJNA953829, PRJNA973939

Research involving human participants, their data, or biological material

Policy information about studies with human participants or human data. See also policy information about sex, gender (identity/presentation), and sexual orientation and race, ethnicity and racism.

Reporting on sex and gender	Sex and gender were not considered as a factor in the data analysis.
Reporting on race, ethnicity, or other socially relevant groupings	Race and socially relevant groupings were not considered as a factor in the data analysis.
Population characteristics	The study utilized publicly available metagenomic and 16S rRNA gene sequencing datasets to investigate age-related and/or mother-to-child transmission of SFB species. Participant information, including age and sampling context, was obtained from the metadata accompanying the publicly available bioproject.
Recruitment	No participant recruitment was conducted for this study. Fecal samples from Mali, The Gambia, and Kenya were obtained from the investigators of the study by Pop et al. (BioProject PRJNA234437) under a Material Transfer Agreement between the Institut Pasteur (Paris, France), the Center for Vaccine Development (Bamako, Mali), the Medical Research Council Unit (Serrekunda, The Gambia), and KEMRI (Kisumu, Kenya).
Ethics oversight	Fecal samples from Mali, The Gambia and Kenya from the study by Pop et al. 45 were obtained by Material Transfer Agreement between Institut Pasteur, Paris, France, and the Center for Vaccine Development, Bamako, Mali, The Medical Research Council Unit, Serrekunda, The Gambia and KEMRI, Kisumu, Kenya. Ethical approval for the fecal sample analysis with participant consent was obtained from the Institutional Review Board under the following Federal Wide Assurance numbers: The Gambia, Medical Research Council Labs FWA 00006873, Kenya Medical Research Institute FWA 00002066, University of Mali Faculty of Medicine Pharmacy and Dentistry FWA 00001769. No additional ethical approval was required for the current work. No participant compensation was given for the current work.

Note that full information on the approval of the study protocol must also be provided in the manuscript.

Field-specific reporting

Please select the one below that is the best fit for your research. If you are not sure, read the appropriate sections before making your selection.

☒ Life sciences ☐ Behavioural & social sciences ☐ Ecological, evolutionary & environmental sciences

For a reference copy of the document with all sections, see nature.com/documents/nr-reporting-summary-flat.pdf

Life sciences study design

All studies must disclose on these points even when the disclosure is negative.

Sample size	We analyzed publicly available bioprojects coomprising16S rRNA gene amplicon sequencing, multiple 16S rRNA gene variable regions, and RNA and DNA metagenomic whole-genome sequencing (WGS). These datasets were obtained from multiple countries and continents. No formal sample size calculation was performed but samples sizes are available in the published manuscript and associated metadata. A search for SFB-positive datasets was continued until there was a decent coverage worldwide and when continents had at least two countries with SFB-positive bioprojects.
Data exclusions	Only bioprojects with 16S rRNA gene amplicon sequencing covering the V1-V2, V4 or V3-V4 16S rRNA gene sequence variable regions were included in the analysis. Analysis was furthermore restricted to 16S rRNA gene amplicon reads ≥200 bp, except for two Bioprojects as described in the Materials and Methods. The V5-V6 16S rRNA gene region, or amplicon reads than 200 bp, could not reliably be assigned to a reference 16S rRNA gene sequence by percent nucleotide identityt due to insufficient sequence variability and were therefore excluded.
Replication	As a quality control of the Human-SFB-ML genome sequences, fecal samples from two individuals from the same geographical region (Mali) were sequenced using metagenomic Hi-C sequencing and independent sequencing platforms (Illumina and PacBio).
Randomization	Randomization was not performed as our analysis was limited to classifying samples/SRAs as segmented filamentous bacteria (SFB) positive or negative based on the detection of any SFB species using read mapping in datasets from multiple independent projects and countries.
Blinding	Blinding was used for the analysis of the presence/absence of SFB in gram stains to ensure that the presence and abundance of SFB-like filaments was in agreement with the 16S rRNA gene amplicon data.

Reporting for specific materials, systems and methods

We require information from authors about some types of materials, experimental systems and methods used in many studies. Here, indicate whether each material, system or method listed is relevant to your study. If you are not sure if a list item applies to your research, read the appropriate section before selecting a response.

Materials & experimental systems

n/a	Involved in the study
☒	☐ Antibodies
☒	☐ Eukaryotic cell lines
☒	☐ Palaeontology and archaeology
☐	☒ Animals and other organisms
☒	☐ Clinical data
☒	☐ Dual use research of concern
☒	☐ Plants

Methods

n/a	Involved in the study
☒	☐ ChIP-seq
☒	☐ Flow cytometry
☒	☐ MRI-based neuroimaging

Animals and other research organisms

Policy information about studies involving animals; ARRIVE guidelines recommended for reporting animal research, and Sex and Gender in Research

Laboratory animals	Germ-free mice of the C57BL/6 genotype were used to propagate Mouse-SFB-NL. No experimentation other than gavage with SFB-positive fecal material was performed on the mice.
Wild animals	No wild animals were used in this study.
Reporting on sex	The sex of the mice was not considered as they were used only for the propagation of Mouse-SFB-NL.
Field-collected samples	Field-collected samples were not part of this study
Ethics oversight	Mouse experiments were performed in accordance with French and European regulations on the protection of the animals used for scientific purposes (Directive 2010/63 of the European Parliament and French decree of February 1, 2013). The mouse experiment (dap210054) was approved by the Institut Pasteur ethical committee for animal experimentation (CETEA, registry number #89) and authorized by the Ministère de l'Enseignement Supérieur et de la Recherche.

Note that full information on the approval of the study protocol must also be provided in the manuscript.

Plants

Seed stocks	Not relevant
Novel plant genotypes	Not relevant
Authentication	Not relevant
